# An aquatic virus exploits the IL6-STAT3-HSP90 signaling axis to promote viral entry

**DOI:** 10.1371/journal.ppat.1011320

**Published:** 2023-04-26

**Authors:** Guoli Hou, Zhao Lv, Wenzhi Liu, Shuting Xiong, Qiushi Zhang, Chun Li, Xiaodong Wang, Liang Hu, Chunhua Ding, Rui Song, Hongquan Wang, Yong-An Zhang, Tiaoyi Xiao, Junhua Li

**Affiliations:** 1 College of Fisheries, Hunan Agricultural University, Changsha, China; 2 State Key Laboratory of Agricultural Microbiology, Hubei Hongshan Laboratory, College of Fisheries, Huazhong Agricultural University, Wuhan, China; 3 Yangtze River Fisheries Research Institute, Chinese Academy of Fishery Sciences, Wuhan, China; 4 Hunan Fisheries Research Institute, Changsha, China; Le Moyne College, UNITED STATES

## Abstract

Viral seasonality in the aquaculture industry is an important scientific issue for decades. While the molecular mechanisms underpinning the temperature-dependent pathogenesis of aquatic viral diseases remain largely unknown. Here we report that temperature-dependent activation of IL6-STAT3 signaling was exploited by grass carp reovirus (GCRV) to promote viral entry via increasing the expression of heat shock protein 90 (HSP90). Deploying GCRV infection as a model system, we discovered that GCRV induces the IL6-STAT3-HSP90 signaling activation to achieve temperature-dependent viral entry. Further biochemical and microscopic analyses revealed that the major capsid protein VP7 of GCRV interacted with HSP90 and relevant membrane-associated proteins to boost viral entry. Accordingly, exogenous expression of either IL6, HSP90, or VP7 in cells increased GCRV entry in a dose-dependent manner. Interestingly, other viruses (e.g., koi herpesvirus, Rhabdovirus carpio, Chinese giant salamander iridovirus) infecting ectothermic vertebrates have evolved a similar mechanism to promote their infection. This work delineates a molecular mechanism by which an aquatic viral pathogen exploits the host temperature-related immune response to promote its entry and replication, instructing us on new ways to develop targeted preventives and therapeutics for aquaculture viral diseases.

## Author summary

Viral seasonality in the aquaculture industry has been an important scientific issue for decades, which is causing billions of economic losses every year worldwide. However, there is a lack of understanding of how temperature influences the pathogenesis of aquatic viruses infecting fish, shellfish, and other ectotherms. Here, deploying aquatic grass carp reovirus (GCRV) as a model system, we demonstrate for the first time that the temperature-dependent IL6-STAT3-HSP90 signaling axis is exploited by GCRV to promote viral entry. We found that HSP90 interacted with viral structural proteins and membrane-associated proteins to enable viral entry. Furthermore, our study indicates other aquatic viruses might evolve a similar mechanism to promote their infection. By elucidating the molecular mechanism of temperature-dependent aquatic viral pathogenesis, our work may help to develop targeted prevention and control strategies against aquatic viral diseases.

## Introduction

Virus-host interaction is a continuous coevolutionary process involving the host developing strategies to limit virus infection and viruses exploiting counter-adaptation mechanisms to survive [1]. As viruses are obligate intracellular parasites, they frequently rely on and utilize host machinery, including the host immune system to facilitate their viral life cycle. It has long been known that many classical immune-surveillance networks, such as NF-kappa B signaling, IL6/STAT3 signaling, autophagy, cytokines, cellular membranes, heat shock proteins (HSPs), and cytoskeletons [1–3], which are supposed to protect the host from viral infections, could be exploited by viruses to ensure their survival. Among them, IL6/STAT3 signaling, an intrinsic antiviral and pro-inflammatory pathway, has been suggested to contribute to several viral disease progression [4,5]. Numerous investigations through blockage or inhibition of key components of IL6/STAT3 signaling have proven the importance of IL6/STAT3 signaling in virus infection [4–7]. Nevertheless, the exact molecular mechanism regarding how the viral life cycle, especially the early phase of viral entry, is governed by IL6/STAT3 signaling remains largely unexplored.

Temperature is one of the key elements regulating all kinds of biological activities. For aquatic ectotherms, temperature not only influences the growth rate of aquatic animals but is also a major determinant of the outbreak of viral diseases. Accordingly, viral diseases in the aquaculture industry show strict seasonality and temperature dependency, which is causing billions of economic losses every year worldwide [8,9]. Thus, uncovering the molecular mechanisms behind this phenomenon is the key to developing targeted prevention and control strategies. Interestingly, IL6/STAT3 signaling is a unique and highly pleiotropic pathway that is evolutionarily conserved and is implicated in both temperature-induced immune responses and virus infection [10,11]. To date, there is no report of the interplay between temperature, IL6/STAT3 signaling, and aquatic virus infection.

Grass carp reovirus (GCRV), a non-enveloped, double-stranded RNA (dsRNA) aquareovirus, is the causative agent of grass carp hemorrhagic disease (GCHD), a lethal, highly contagious, and notifiable disease in grass and black carp farming industry, which suffered heavily each year by the seasonal viral disease [12]. Decades of epidemiological, experimental, and practical studies have demonstrated that the optimum temperature of GCHD is around 25–28°C [13]. Virus entry into animal cells, the first step to launch a disease, is initiated by attachment to receptors and followed by penetration through (non-enveloped viruses) or fusion with (enveloped viruses) cellular membranes [14]. Pioneering studies have suggested that tight crosstalk between viral structure proteins (e.g., VP5, VP7, VP35) and host machinery (e.g., endosome, microtubules, dynamin, receptors, HSPs) are involved in the viral entry of GCRV infection [15–21]. However, our understanding of the temperature-dependent GCRV pathogenesis is still in its infancy. In this study, we employed the infection of grass carp by GCRV as a homologous virus-host model to dissect the intricate molecular mechanism behind seasonality and temperature dependency of virus infection in aquatic ectotherms. We discovered that GCRV exploited the IL6-STAT3-HSP90 axis to enable temperature-dependent viral entry. Mechanistically, major capsid protein VP7 of GCRV interacted with HSP90 and relevant membrane-associated proteins to promote viral entry. Exogenous expression of either HSP90 or VP7 in cells rendered an increased viral entry therefrom. Importantly, we found that such mechanism may be conserved in many other aquatic viruses, including DNA or RNA viruses infecting ectothermic vertebrates. Overall, our work delineated for the first time a pivotal role of IL6-STAT3-HSP90 axis in the temperature-dependency of aquatic viral entry.

## Results

### Temperature-dependency phenotype of GCRV infection

**Fig 1:**
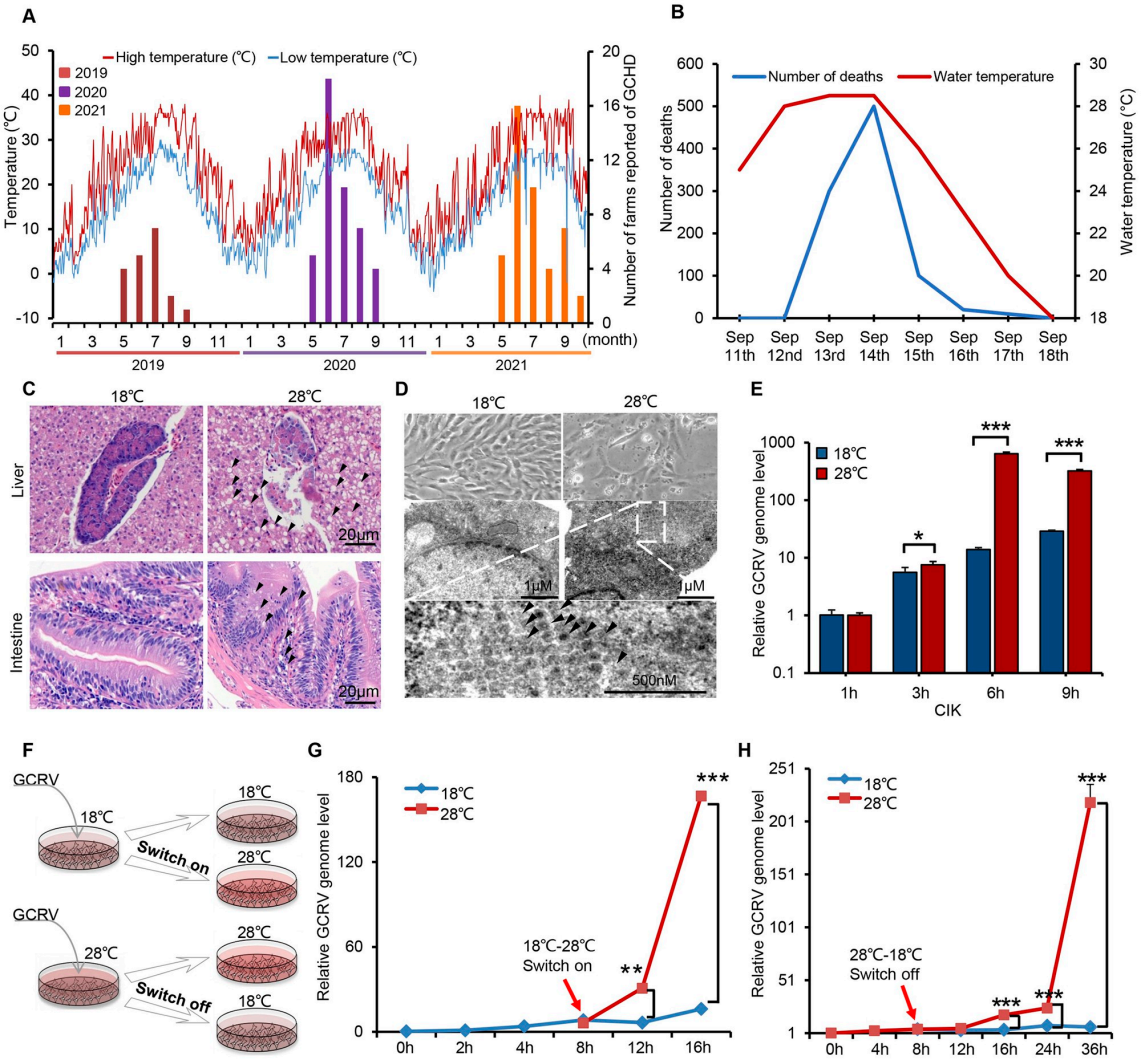
Temperature dependency of GCRV infection. (**A**) Double-axis graph depicting the GCHD survey results from 46 fisheries in Hunan province in the last three years (2019–2021). The historical weather data for daily high (red line) and low (blue line) temperatures and the statistics of GCHD outbreaks were recorded on the left y-axis and right y-axis, respectively. (**B**) A double-axis graph depicting a natural-onset of GCHD outbreak in fish ponds in mid-September was conducted. The number of deaths (blue line) and water temperature (red line) were recorded on the left y-axis and right y-axis, respectively. (**C**) Grass carp were challenged with GCRV-AH528 (100 μL at 1.0 × 10^6^ TCID_50_ mL^-1^ per fish) by intraperitoneal injection at different temperatures. Liver and intestine by day 5 post-infection were collected for HE staining assay. The black arrow denotes vacuolization. Scale bar = 20 μm. (**D**) The Cytopathic Effect (CPE) and virus particles were observed by optical microscope and transmission electron microscope in CIK cells infected with GCRV (MOI = 5) under different temperatures (18°C, 28°C). Scale bar = 500 μm. (**E**) The relative GCRV genome replication from infected CIK cells under different temperatures (18°C, 28°C) was analyzed by RT-PCR with the VP4 primer pair. (**F**) Schematic of experimental design showing temperature-switch affects GCRV replication in CIK cells. (**G**) The relative GCRV genome replication from 18°C of infection switch to 28°C of infection was analyzed by RT-PCR. (**H**) The relative GCRV genome replication from 28°C of infection switch to 18°C of infection was analyzed by RT-PCR with the VP7 primer pair. Data were presented as mean ± SD from three independent experiments. Statistical analysis was performed using one-way ANOVA between different groups and the asterisk (*) indicates significant differences between groups. * *p*<0.05, **p<0.01, ***p<0.001.

Compared with endotherms, viral diseases in lower ectotherms are more susceptible to temperature fluctuation. To explore the relationship and mechanism between the seasonality of aquatic viral disease and temperature, we first surveyed grass carp hemorrhagic disease (GCHD), which is most likely caused by GCRV infection. The data showed that from summer to autumn (May to September) in the south of China, when mean temperature and diurnal variation tend to be high, is a peak period of hemorrhagic disease incidence (Figs 1A and S1A), suggesting that temperature is the key determinant of GCHD outbreak. Additionally, we documented a natural onset of GCHD outbreak in the cultured ponds under natural climatic conditions in mid-September. As shown in Fig 1B, the water temperature increased by about 3 degrees from 11^th^ to 14^th^ (25°C to 28°C), when GCHD began and the number of deaths surged to 500 by 14^th^ Sep. Typical symptoms of hemorrhage and inflammation were observed on the fins, skins, and intestines of hemorrhagic carp (S1B Fig). From 14^th^ to 18^th^, the water temperature, as well as the number of deaths, declined sharply due to rainy weather (Fig 1B). The mortality curve of grass carp coincided well with the temperature variation curve, with the number of deaths peaked at around 28°C and recovered from the disease outbreak at around 18°C (Fig 1B), suggesting that temperature or temperature stress may play an important role during the outbreak and alleviation of GCRV disease, which is a common occurrence in viral diseases of aquaculture [8,9,13]. To dissect the molecular mechanism underlining the temperature dependency of GCRV infection, we performed the *de novo* GCRV infection at 18°C and 28°C to represent resistant infection and permissive infection, respectively. RT-PCR analysis showed that the relative viral genome level in tissues from 28°C of infection was much higher compared with tissues from 18°C of infection (S1C Fig). Moreover, hematoxylin and eosin (H&E) staining and pathological analysis showed that pronounced vacuolization, which was associated with inflammation, was observed in liver and intestine tissue sections from 28°C of infection (Fig 1C). Besides that, electron microscopy analysis of GCRV infected grass carp (*Ctenopharyngodon idellus*) kidney (CIK) cells showed that viral infection at 28°C displayed a more typical cytopathogenic effect (CPE) and more virus particles array within cells compared with 18°C of infection (Fig 1D). Consistent with the electron microscopic data, RT-PCR analysis showed that the viral proliferation rate at 28°C was almost two orders of magnitude faster than 18°C in CIK cells (Fig 1E). Similar results were obtained from grass carp ovary (GCO) cells infected at different temperatures (S1D Fig). These data collectively indicated that temperature plays an important role in GCRV pathogenesis. To determine how temperature impact GCRV proliferation, we conducted a temperature-switch experiment. We first infected CIK cells at 18°C or 28°C for 8 h, then switched the temperature to 28°C or 18°C, respectively (Fig 1F). RT-PCR analysis showed that virus proliferation from 18°C to 28°C switches surged by 10-fold (Fig 1G), while 28°C to 18°C switches significantly slowed the viral genome replication by a factor of 5 to 32 (Fig 1H), confirming the vital role of temperature on viral infection and proliferation. Collectively, these results support the conclusion that the epidemiology and pathology of GCRV infection show a temperature-dependent phenotype.

### Temperature dependency of GCRV infection counts on the activation of IL6/STAT3 signaling

**Fig 2:**
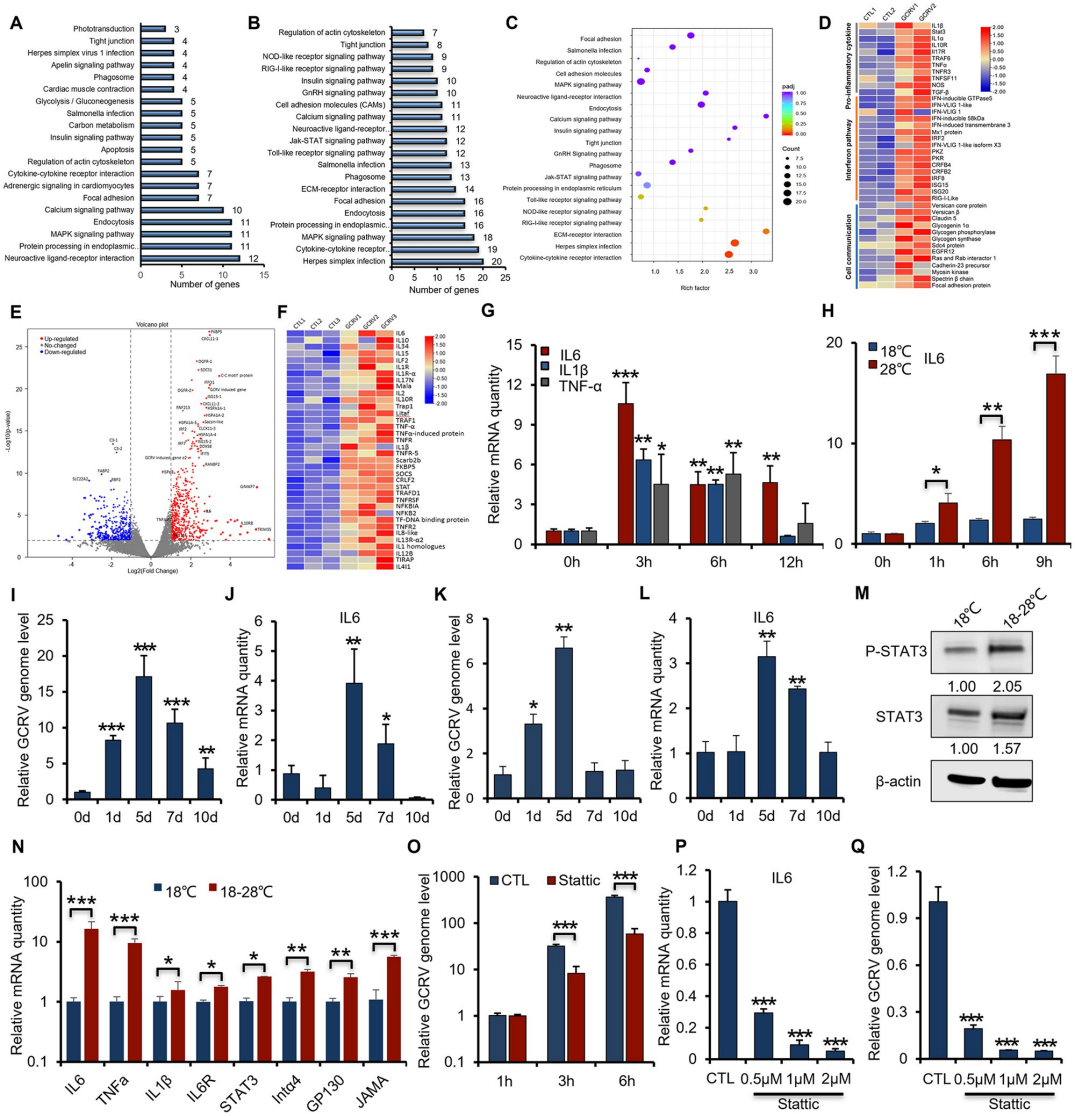
IL6-STAT3 signaling pathway mediates temperature-dependent GCRV infection. (**A-B**) KEGG pathway enrichment analysis of DEGs from transcriptomic data related to CIK cells by temperature-switch treatment from 18°C to 28°C (NCBI SRA database accession number PRJNA862271) (A) and grass carp head kidney by GCRV infection (NCBI SRA database accession number PRJNA759556) (B). The top 20 significantly enriched pathways (ranked by *p*-value) are shown in the bar graph. (**C**) KEGG pathway enrichment analysis of transcriptomic data from grass carp head kidney by GCRV infection at 28°C was presented in the form of scatterplots based on enrichment factor and p-value. The enrichment factor is the ratio between the DEG number and the number of all genes in a certain gene enrichment term. The sizes of the dots denote the number of DEGs, while colors correspond to the p-value. (**D**) Heat map analysis of DEGs that were up-regulated in the GCRV-infected spleen tissue was summarized (NCBI SRA database accession number PRJNA759556). (**E**) Transcriptomic data of all genes differentially regulated from GCRV-infected spleen samples of grass carp (NCBI SRA database accession number PRJNA600033) were analyzed by volcano plot analysis. (**F**) Transcriptomic data from (E) profiling some upregulated pro-inflammatory genes were analyzed by heat map analysis. (**G**) CIK cells under temperature-switch treatment from 18°C to 28°C were harvested to analyze the expression of pro-inflammatory genes by RT-PCR. (**H**) CIK cells infected with GCRV (MOI = 5) under different temperatures (18°C, 28°C) were harvested to quantify the expression of IL6 by RT-PCR. (**I-L**) The relative proliferation of the GCRV genome (with the VP4 primer pair) and expression of IL6 in the spleen (I-J) and gills (K-L) of grass carp from different time points were quantified by RT-PCR. (**M-N**) CIK cells under temperature-switch treatment from 18°C to 28°C for 1 h were harvested to analyze the signal of STAT3 and the IL6-STAT3 axis transcription by Western blotting analysis (**M**) and RT-PCR (**N**), respectively. STAT3 phophorylation specific antibody (Y705) was probed to dictate the activation of STAT3. The density of the Western blot bands was quantified using ImageJ software. (**O**) CIK cells pre-treated with STAT3 inhibitor Stattic (1μM) were infected with GCRV (MOI = 5) at 28°C and harvested to analyze the relative viral genome level by RT-PCR with the VP7 primer pair. (**P-Q**) CIK cells pre-treated with different doses of STAT3 inhibitor Stattic (0.5 μM, 1 μM, 2 μM) were infected with GCRV (MOI = 5) at 28°C and harvested to analyze the expression of IL6 (P) and relative viral genome level (O) by RT-PCR with the VP7 primer pair. Data were presented as mean ± SD from three independent experiments. Statistical analysis was performed using one-way ANOVA between different groups and the asterisk (*) indicates significant differences between groups. * *p*<0.05, **p<0.01, ***p<0.001.

To delineate the mechanism of temperature dependency of GCRV infection, we hypothesized that the temperature-related immune responses may be exploited by the virus to promote its infection efficiency and propagation later on. We thus attempted to conduct unbiased screening based on two transcriptomic analyses of temperature stress and GCRV infection to explore the genes and pathways related to temperature-dependent GCRV pathogenesis. We first carried out a transcriptomic analysis of CIK cells (without virus infection) under temperature-switch treatment from 18°C to 28°C to probe the possible pathways involved in temperature-related responses (Fig 1F). KEGG enrichment analysis revealed that, among the top 20 enriched pathways (ranked by *p*-value) for temperature-switch treatment differentially-regulated genes, those 1808 genes were preferentially enriched in pathways related to dealing and communicating with environmental stimuli, such as focal adhesion, cytokine-cytokine receptor interaction pathway, calcium signaling pathway, endocytosis, and protein processing in the endoplasmic reticulum (Fig 2A). After then, we performed a transcriptomic analysis of grass carp head kidney infected with GCRV at 28°C to probe the enriched pathways related with GCRV infection. Interestingly, KEGG enrichment analysis confirmed that these pathways as shown above, together with canonical antiviral signaling pathways, such as Toll/RIG-I/NOD-like signaling pathways, JAK-STAT signaling pathway, and Herpes simplex infection pathway, were significantly enriched (Fig 2B). Further scatterplot of KEGG pathway enrichment analysis of differentially expressed genes (DEGs) for GCRV infected head kidney (ranked by *p*-value) revealed that cytokine-cytokine receptor interaction pathway was one of the most significant enriched pathways (Fig 2C). Besides, heat map analysis of transcriptomic data from GCRV infected grass carp spleen tissue further demonstrated that pro-inflammatory cytokines (e.g., IL1β, STAT3, IL17, IL1R, TNF-α, NOS2), interferon related genes (e.g., ISG58, IFN-VLIG 1, ISG20, ISG15, IFITM3, Mx1, PKR, PKZ), and cell-to-cell interaction related genes (e.g., cadherin-23, claudin-5, glycogen, focal adhesion protein, EGFR) were significantly up-regulated in GCRV infected spleen (Fig 2D). Also, heat map analysis of grass carp head kidney infected with GCRV showed similar results (S2A Fig), that pro-inflammatory response, interferon pathway, and cell communication related pathways were highly involved in GCRV infection. In addition, the data mining of transcriptomic data from GCRV-infected spleen tissues by volcano plot and heat map analyses were again consistent with our results, pro-inflammatory genes were highly involved in the pathogenesis of GCRV infection (Fig 2E and 2F) [22]. Thus, transcriptomic analyses of temperature-switch and GCRV infection conjointly demonstrated that pro-inflammatory cytokines may be involved in temperature-dependent GCRV pathogenesis, In light of the pleiotropic roles of pro-inflammatory, pyrogenic cytokines, such as IL6, IL1β, and TNF-α, in both viral infection and temperature-related inflammation. In addition, it is reported that circulating IL6 from peripheral sites could act as a messenger that transmits inflammatory information to the central nervous system (CNS) to mediate temperature-related responses through IL6-STAT3 signaling [11,23,24]. We thus hypothesized that these pyrogenic cytokines, particularly IL6-mediated signaling pathways may be exploited by viruses to augment viral propagation. Indeed, RT-PCR analysis of the gill samples from *de novo* GCRV-infected grass carp showed that higher expression of IL6, IL1β, and TNF-α correlated well with faster GCRV proliferation at 28°C (S1C and S2B Figs). To determine and validate the role of temperature on viral infection and IL6/STAT3 signaling, we firstly evaluated the effect of temperature-switch on the immune responses of grass carp tissues or CIK cells. In the absence of viral infection, a temperature switch from 18°C to 28°C for CIK cells, or grass carp in the fish tank, significantly induced the expression of IL6, IL1β, and TNF-α (Figs 2G, S2C and S2D). The elevation of IL6 was the most evident, as it reached the peak rapidly in CIK cells (Fig 2G), gills, and intestines of grass carp (S2C and S2D Fig). Consistently, GCRV infection in CIK cells induced the expression of pyrogenic cytokines (IL6, IL1β, TNF-α) and plasma membrane receptors (gp130, IL6R, integrin-α4, JAMA) in a temperature-dependent manner (Figs 2H, S2E and S2F). Notably, asymptomatic infection of GCRV at 18°C barely induced the expression of IL6, while the higher temperature of 28°C infections significantly induced their expression (Fig 2H), suggesting that GCRV exploits IL6 signaling to facilitate its infection at 28°C. Besides, the transcription of Hif-1α, which is commonly related to hyperthermia and hypoxia stimulus and might be linked with the fish disease outbreak in aquaculture, remained nearly unaffected in CIK cells upon GCRV infection at different temperatures (S2G Fig), suggesting that pro-inflammatory cytokines might be the major factor involved in GCRV infection. In addition, RT-PCR analysis of spleen and gill samples from grass carp artificially infected by GCRV further confirmed that the replication of GCRV genome in the spleen (Fig 2I and 2J) and gills (Fig 2K and 2L) paralleled with induced expression of IL6, signifying our hypothesis that the expression of IL6 may facilitate GCRV infection. As STAT3 is a transcription factor downstream of IL6 signaling activation, we then examined whether IL6/STAT3 axis mediates the temperature-dependency of GCRV infection. By Western blotting analysis with STAT3 activation specific antibody (P-STAT3, Y705), we confirmed that temperature-switch activated STAT3 and increased the protein level of STAT3 (Fig 2M). RT-PCR analysis further demonstrated that temperature-switch greatly induced the transcription of IL6/STAT3 signaling axis-related genes (IL6, IL6R, gp130, STAT3, IL1β, TNF-α) and membrane-related genes (integrin-α4, JAMA) (Fig 2N). To examine whether STAT3 activation is essential for GCRV infection, we deployed a STAT3-specific inhibitor Stattic to treat cells [25]. As shown in Fig 2O, Stattic treatment greatly reduced the replication of GCRV by 86%. Moreover, Stattic treatment impaired the transcription of IL6, IL1β, TNF-α, and GCRV genome replication (Figs 2P, 2Q, and S2H) in a dose-dependent manner. Consistently, Western blotting analysis demonstrated that Stattic treatment in CIK cells greatly impaired the activation of STAT3, as shown by the P-STAT3 signal (S2I Fig). While the cell viability assay showed that Stattic had a negligible cytotoxic effect on the cells under our experimental conditions (S2J Fig). Altogether, these data indicate that IL6/STAT3 signaling activation is essential for the temperature-dependency of GCRV pathogenesis.

### IL6-STAT3-HSP90 axis mediates temperature dependency of GCRV infection

**Fig 3:**
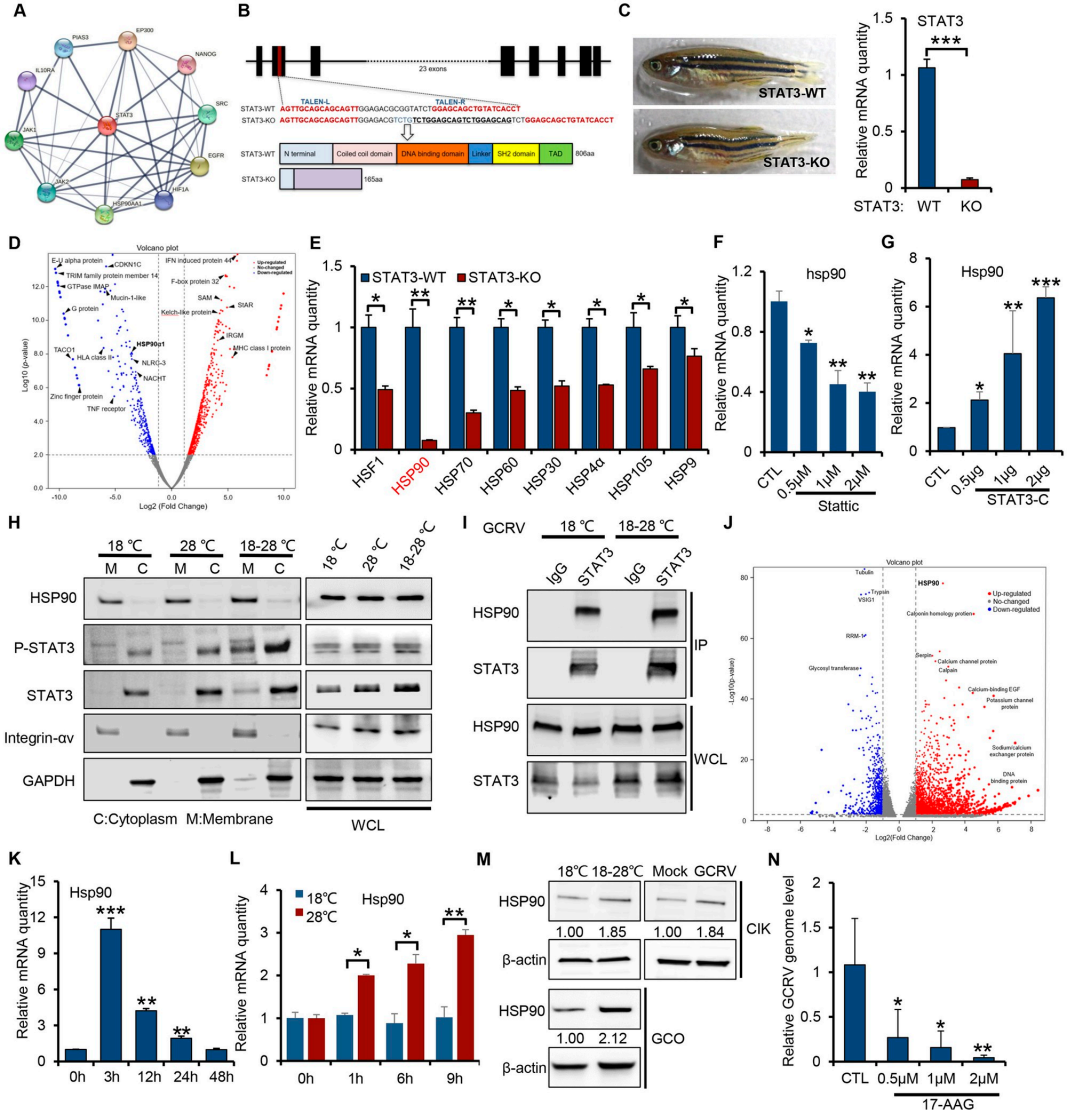
IL6-STAT3-HSP90 axis mediates temperature dependency of GCRV infection. (**A**) Protein-protein interaction network of human STAT3 was analyzed online with the STRING database (http://string-db.org) under a ‘Creative Commons BY 4.0’ license (https://cn.string-db.org/cgi/access?footer_active_subpage=licensing). (**B**) Schematic diagram of constructing zebrafish STAT3 knockout mutant. (**C**) STAT3 gene expression in the STAT3-KO mutant was verified by RT-PCR. (**D**) Transcriptomic data of all genes differentially regulated from STAT3-KO mutant were collected to quantify the differentially regulated genes by volcano plot analysis (raw data in NCBI database accession number SRR3985375). (**E**) The expression of heat shock proteins from STAT3-WT and STAT3-KO mutants was validated by RT-PCR analysis. (**F**) CIK cells treated with different doses of STAT3 inhibitor Stattic (0.5 μM, 1 μM, 2 μM) were prepared to quantify the relative expression of HSP90 by RT-PCR analysis. (**G**) CIK cells transfected with different doses of STAT3-C plasmids were prepared to quantify the transcription of HSP90 by RT-PCR analysis. (**H**) CIK cells under different temperatures (18°C, 28°C) or temperature-switch treatment from 18°C to 28°C were prepared to analyze the subcellular fractionation of HSP90 and STAT3 by Western blotting. Integrin-αv and GAPDH served as the marker of the plasma membrane, and cytoplasm, respectively. Whole-cell-lysates were loaded as controls. (**I**) CIK cells under temperature-switch treatment from 18°C to 28°C were prepared for endogenous co-immunoprecipitation analysis with STAT3 antibody to examine the interaction between HSP90 and STAT3. IP and WCL indicate immunoprecipitation and whole-cell-lysates, respectively. (**J**) Volcano plot analysis of all genes differentially regulated from transcriptomic data in CIK cells under temperature-switch treatment from 18°C to 28°C. (**K**) Intestines of grass carp under temperature-switch treatment from 18°C to 28°C were prepared to quantify the relative expression of HSP90 by RT-PCR. (**L**) CIK cells infected with GCRV (MOI = 5) under different temperatures were prepared to analyze the relative expression of HSP90 by RT-PCR. (**M**) CIK/GCO cells under temperature-switch treatment from 18°C to 28°C or GCRV infection (MOI = 5) at 28°C were prepared to analyze the expression of HSP90 by Western blotting analysis. The density of the Western blot bands was quantified using ImageJ software. (**N**) CIK cells pre-treated with different doses (0.5 μM, 1 μM, 2 μM) of HSP90 inhibitor 17-AAG were infected with GCRV (MOI = 5) at 28°C and harvested to quantify the relative viral genome replication with the VP7 primer pair. Data were presented as mean ± SD from three independent experiments. Statistical analysis was performed using one-way ANOVA between different groups and the asterisk (*) indicates significant differences between groups. * *p*<0.05, **p<0.01, ***p<0.001.

STAT3 is a signal transducer and transcription activator that is critically involved in cellular pro-survival processes such as proliferation, differentiation, and apoptosis [26,27]. To determine the key downstream effectors of IL6-STAT3 signaling in the temperature-dependent GCRV pathogenesis, we first performed a protein-protein interaction network analysis of human STAT3 with the STRING database (http://string-db.org), which showed that STAT3, in the form of a hub protein with a high node degree, actively associated with many molecules such as JAK1/2, IL10RA, EP300, NANOG, EGFP, HIFA, and HSP90AA (Fig 3A). Among them, HSP90AA1 belongs to the HSP90 family and is one of the most important molecular chaperones evolved to maintain functionally active of client proteins under both physiological and temperature stress conditions such as cancers, heat shock, and virus infection [28]. It has been previously reported that HSP90, together with IL6/STAT3 signaling, is essential for the progression of various diseases, infections, and cancers [29–32]. Thus, we postulated that IL6/STAT3 signaling might regulate HSP90 expression to engage in viral infection. To probe the role of HSP90 in the IL6-STAT3 signaling pathway, we first generated STAT3 deficient zebrafish mutant by TALEN-based genome editing technology (Fig 3B and 3C). STAT3-KO mutant zebrafish codes for a truncated protein containing only 46 aa of the N-terminus of STAT3-WT (Fig 3B). Volcano plot analysis showed that, compared with STAT3-WT, the expression of HSP90 (refers to HSP90α in full text) and other immune-related genes in STAT3-KO mutant zebrafish were significantly differentially expressed (Fig 3D). RT-PCR analysis further confirmed that multiple heat shock proteins in STAT3-KO mutant were greatly reduced. Importantly, the STAT3-KO mutant almost abolished the expression of HSP90 (Fig 3E), suggesting that HSP90 is a downstream target gene of STAT3 signaling. Consistently, STAT3 inhibition by Stattic treatment in CIK cells dampened the expression of HSP90 in a dose-dependent manner (Fig 3F). To minimize the possibility of off-target effects of Stattic [33], we deployed STAT3-C (A661C, D663C), a constitutively active mutant of STAT3 [34], to examine the regulatory role of STAT3 on HSP90. As shown in Figs 3G, S3A and S3B, overexpression of STAT3-C in CIK cells upregulated the transcription of IL6, IL1β, TNF-α and HSP90 in a dose-dependent manner, which concomitantly promoted the GCRV infection, indicating that HSP90 is a downstream target gene of STAT3 signaling that is vital for GCRV infection. Similar results were found in 293T cells transfected with STAT3-C (S3C Fig). Besides, STAT3 knockdown with siRNA transfection further confirmed that STAT3 regulates the transcription of IL6, IL1β, TNF-α, HSP90, and is essential for GCRV infection (S3D and S3G Fig). In addition, immunofluorescence and fractionation analysis showed that HSP90 is mainly located on the plasma membrane in resting cells, while STAT3 is mainly located in the cytoplasm (Figs 3H and S3H). Temperature-switch from 18°C to 28°C in CIK cells induced the activation of STAT3 (as shown by increased P-STAT3 signal) and its trafficking from the cytoplasm to the cellular membrane fraction where HSP90 is located (Fig 3H), suggesting that temperature stress induced the association between STAT3 and HSP90. Similarly, endogenous co-immunoprecipitation analysis from CIK cells under temperature-switch treatment confirmed that STAT3 bounds to HSP90 (Fig 3I). To probe the role of HSP90 in the temperature-dependency of GCRV infection, we conducted a volcano plot analysis from the transcriptomic data of CIK cells under temperature-switch treatment, showing that HSP90 was significantly induced by temperature-switch treatment (Fig 3J). RT-PCR analysis of intestines, gills, and CIK cells from temperature-switch treatment further confirmed that the transcription of HSP90 was induced (Figs 3K, S3I and S3J). In addition, RT-PCR analysis of CIK cells further showed that GCRV infection induced the expression of HSP90 in a temperature-dependent manner, from which asymptomatic infection of GCRV at 18°C barely induced the expression of HSP90, while the higher temperature of 28°C infections significantly induced its expression (Fig 3L), suggesting that GCRV may exploit the expression HSP90 to facilitate viral infection at 28°C. The Western blotting analysis further validated the elevated protein level of HSP90 under a temperature-switch from 18°C to 28°C or GCRV infection at 28°C (Fig 3M). Finally, CIK cells treated with 17-AAG, an HSP90-specific inhibitor [35], greatly blocked the proliferation of GCRV in a dose-dependent manner (Fig 3N). Collectively, these data indicate that HSP90, a downstream molecule of STAT3 signaling, is associated with STAT3 and plays a vital role in the temperature dependency of GCRV infection.

### IL6-STAT3-HSP90 axis regulates GCRV entry

**Fig 4:**
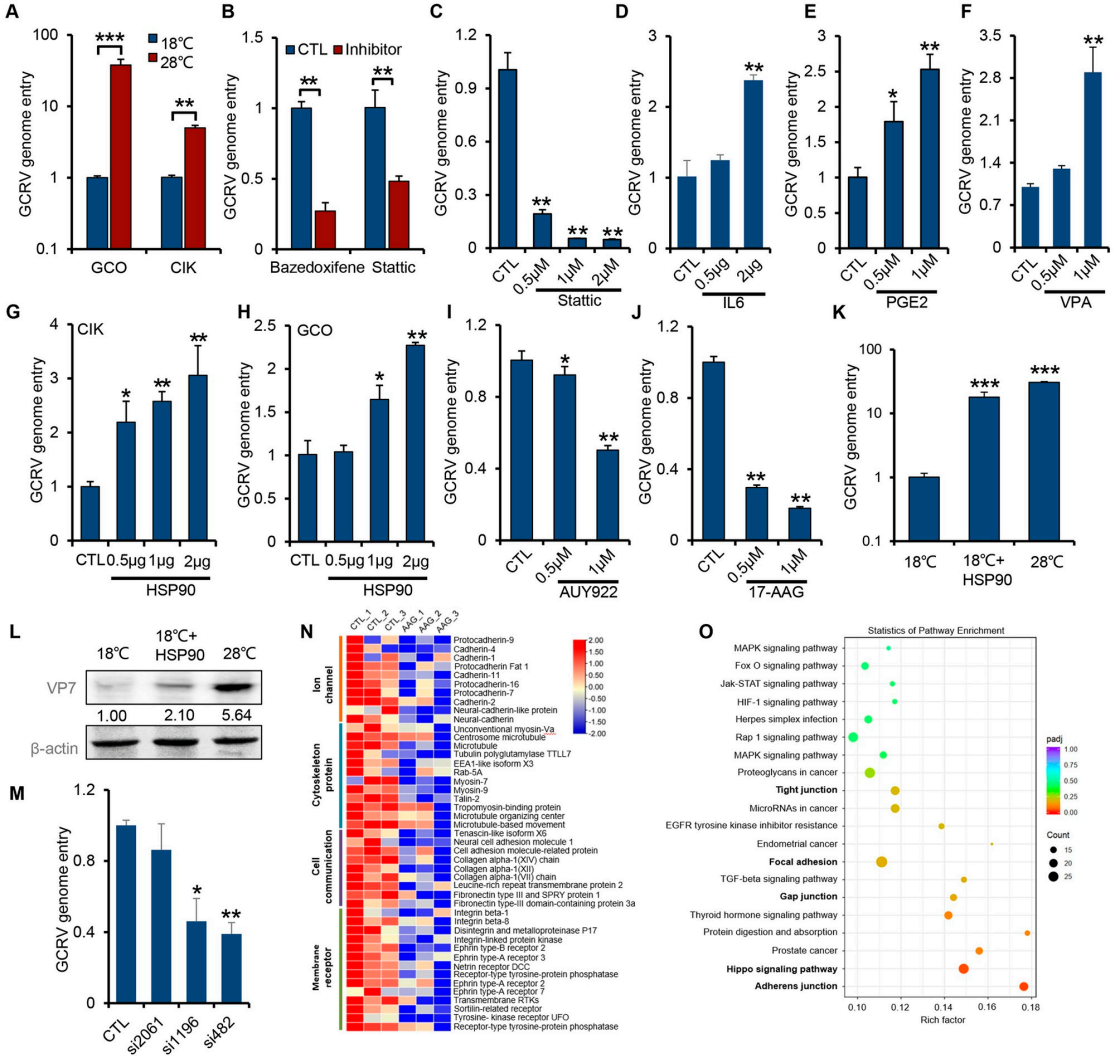
IL6-STAT3-HSP90 axis regulates GCRV entry. (**A**) GCO/CIK cells infected with GCRV (MOI = 5) at different temperatures for 1 h were prepared to quantify the relative genome entry by RT-PCR with the VP7 primer pair. (**B**) CIK cells pre-treated with 1 μM of bazedoxifene or Stattic were infected with GCRV (MOI = 5) at 28°C followed by relative GCRV genome entry quantification by RT-PCR with the VP7 primer pair. (**C-F**) CIK cells pre-treated with different doses of Stattic, IL6 (plasmid transfection), PGE2, or VPA were infected with GCRV (MOI = 5) at 28°C for 1 h and harvested to analyze the relative viral genome entry by RT-PCR analysis with the VP7 primer pair. (**G-H**) CIK or GCO cells transfected with different doses of HSP90 plasmids were infected with GCRV (MOI = 5) for 1 h and harvested to quantify the relative viral genome entry by RT-PCR with the VP7 primer pair. (**I-J**) CIK cells pretreated with different doses (0.5 μM, 1 μM, 2 μM) of AUY922 or 17-AAG were infected with GCRV (MOI = 5) at 28°C for 1 h and harvested to quantify the relative viral genome entry by RT-PCR with the VP7 primer pair. (**K-L**) CIK cells grown at 18°C, 28°C, or 18°C pre-incubated with exogenous HSP90 were infected with GCRV (MOI = 5) for 1 h and harvested to analyze the relative viral entry level by RT-PCR (K) and Western blotting (L). (**M**) CIK cells transfected with control siRNA and three different HSP90 siRNAs were infected with GCRV (MOI = 5) for 1 h, followed by harvest for viral genome entry quantification by RT-PCR. (**N-O**) Transcriptomic data of CIK cells treated with 17-AAG (raw data in NCBI database accession number PRJNA862332) were prepared to analyze the differentially downregulated genes by heat map (N) and KEGG enrichment (O) analysis. Data were presented as mean ± SD from three independent experiments. Statistical analysis was performed using one-way ANOVA between different groups and the asterisk (*) indicates significant differences between groups. * *p*<0.05, **p<0.01, ***p<0.001.

To determine which step of the viral life cycle is governed by the IL6-STAT3-HSP90 axis, we first examined the viral genome copy number in CIK and GCO cells within 1 h of infection, which is supposed to be the entry period for viral genome [36]. RT-PCR analysis showed that compared with 18°C of infection, infection at 28°C in CIK or GCO cells increased the intracellular viral genome number by 5- to 40-fold (Fig 4A), suggesting that temperature plays an important role in the infectivity of GCRV. Given that HSP90 was reported to function as a co-receptor or key chaperon for viral entry [37–39], we hypothesized that IL6-STAT3-HSP90 signaling was exploited by the virus for establishing a successful infection during the initial step and launching a viral disease later on.

To test this, we employed several activators or inhibitors downstream IL6-STAT3 signaling to treat cells for 2 h, then infected cells for 1 h with GCRV to quantify viral entry (S4A Fig). We first tested the cytotoxicity of the drugs in CIK cells after treated for 2 h. The cell viability assay showed that the drugs under our experimental conditions had a negligible cytotoxic effect on the cells (S4B Fig). RT-PCR analysis showed that treating cells with inhibitors including bazedoxifene [40] or Stattic [25], diminished the infection efficiency of GCRV by 50% to 70% (Fig 4B and 4C). While IL6-STAT3 signaling activators, including IL6 [10], prostaglandin E2 (PGE2), or valproic acid (VPA) [41] significantly increased the infection efficiency and the IL6 transcription (Figs 4D–4F, S4C and S4D). Both activators and inhibitors appeared to have a dose-dependent effect on the viral infection (Fig 4C–4F). Next, we determined whether HSP90 regulates GCRV entry. We found that overexpression of HSP90 in CIK or GCO cells increased the viral entry in a dose-dependent manner, as shown by the RT-PCR analysis (Figs 4G, 4H, and S4E). In addition, we found that the plasma membrane-associated proteins, such as integrin-α4, LamR, JAMA, which are likely the bona fide receptors for GCRV entry, are significantly induced by HSP90 overexpression (S4F Fig). On the contrary, HSP90 inhibition by two inhibitors, including AUY922 [42] and 17-AAG [35], strongly impaired the viral entry (Fig 4I and 4J). In addition, except for the inhibitory effect on viral entry, those inhibitors to the IL6-STAT3-HSP90 axis also displayed a parallel effect on viral genome replication (S4G Fig) and propagation thereafter as shown by the CPE monitoring (S4H Fig). To examine whether HSP90 could confer an increased viral entry of GCRV infection at low temperature, we incubated exogenous HSP90 into CIK cells grown at 18°C followed by GCRV infection, RT-PCR analysis showed that adding HSP90 into CIK cells grown at 18°C significantly increased the viral entry to a level comparable with CIK cells grown at 28°C (Fig 4K). The Western blotting analysis further confirmed the increased structural protein level of viral entry (Fig 4L), suggesting that HSP90 is associated with membrane protein and plays a crucial role in mediating GCRV entry. Consistently, HSP90 knockdown with siRNA transfection further confirmed that HSP90 is involved in GCRV entry (Figs 4M and S4I). To further dissect the role of HSP90 in viral infection, we performed a transcriptomic analysis of CIK cells treated with HSP90 specific inhibitor 17-AAG [35], we found that the transcription of membrane receptors, cell communication, cytoskeleton protein, and ion channel-related proteins were significantly downregulated when HSP90 was inhibited (Fig 4N). Further KEGG enrichment analysis of all the down-regulated genes showed that adhesion and junction-related pathways (e.g., tight junction, focal adhesion, gap junction, Hippo signaling pathway, adherens junction) were the most significantly enriched pathways (Fig 4O), which mainly function as bridges for cell-to-cell and cell-to-environment communications. Combining these results with primary plasma membrane localization of HSP90 by previous data, it is conceivable that HSP90 mainly locates at the plasma membrane of CIK cells to function as a co-receptor or key chaperon, binding to bona fide viral receptors and structural proteins of GCRV to facilitate initial viral infection.

### HSP90 interacts with VP7 of GCRV to promote viral entry

**Fig 5:**
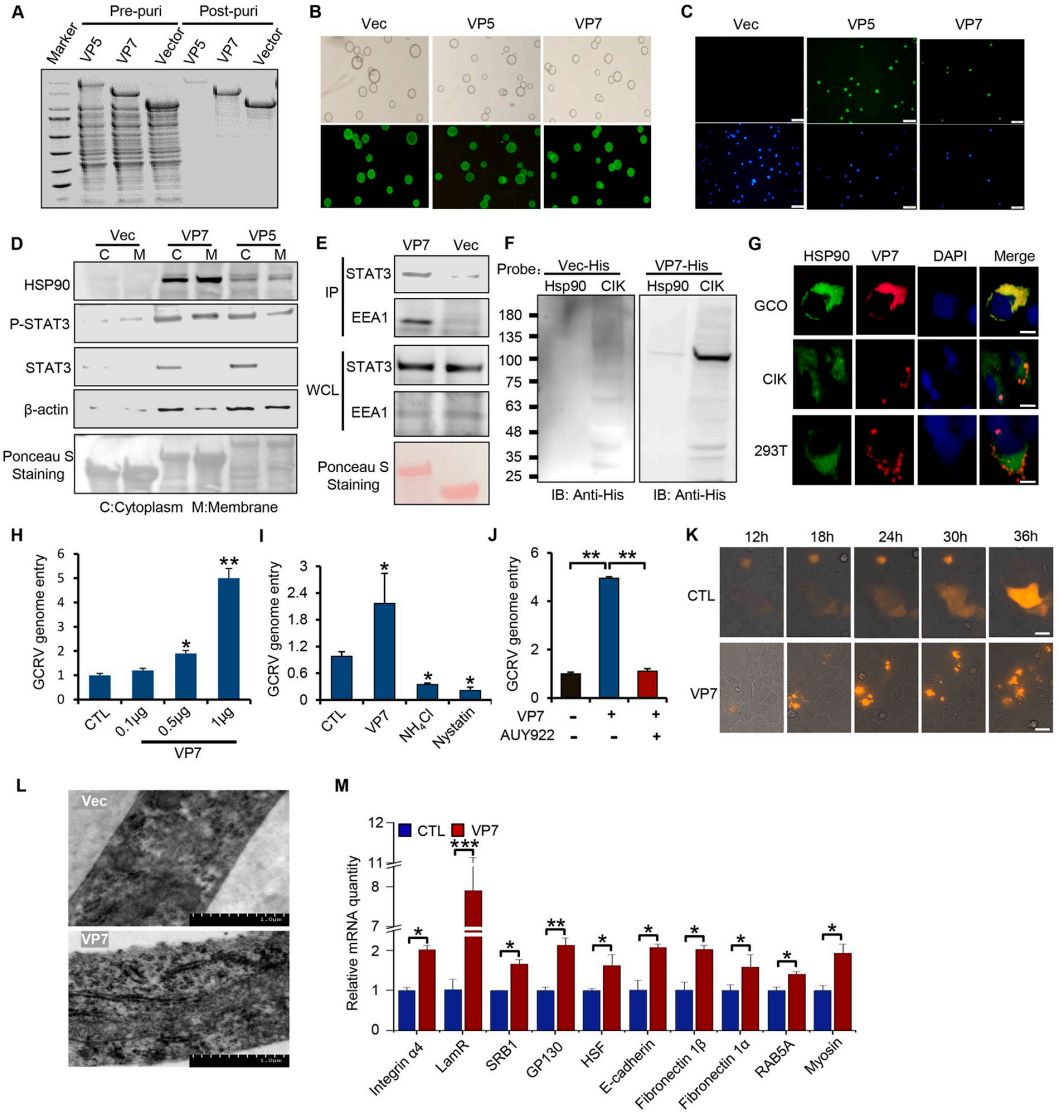
HSP90 interacts with VP7 and STAT3 on the cellular membrane to promote GCRV entry. (**A**) Recombinant protein VP5-HIS-EGFP and VP7-HIS-EGFP were purified using standard nickel-column affinity protocol to homogeneity and analyzed by SDS-PAGE. (**B**) Purified recombinant protein coupled with Ni beads was examined by optical microscopy analysis. (**C**) GCO cells were incubated with the purified recombinant protein VP5-HIS-EGFP /VP7-HIS-EGFP, and the binding between VP5/VP7 and GCO cells was analyzed by optical microscopy analysis. The empty vector was handled as a control. Scale bars denote 5 μm. (**D**) CIK cells were prepared for subcellular fractionation followed by pulldown analysis with purified his-tagged VP7/VP5. The precipitates were analyzed by Western blotting with the corresponding antibody. Ponceau S Staining served as a loading control. (**E**) CIK cell lysates were incubated with purified his-tagged VP7, The precipitates from the pulldown complex were analyzed by Western blotting with the corresponding antibody. EEA1 (early endosomal antigen 1) served as an early endosome marker. Ponceau S Staining served as a loading control. Pulldown analyses were conducted to determine the interaction between STAT3 and VP7 in CIK cells. IP and WCL indicate immunoprecipitation and whole-cell-lysates, respectively. (**F**) The direct interaction between VP7 and HSP90 was examined by far-Western blotting analysis *in vitro*. His tagged VP7 served as bait followed by his antibody to probe the HSP90 signal from the CIK lysates. (**G**) GCO, CIK, or 293T cells stably expressing tagged VP7 and HSP90 were prepared to analyze their colocalization by immunofluorescence analysis. Scale bars: 20 μm. (**H**) CIK cells pre-treated with different doses of VP7 were infected with GCRV at 28°C for 1 h and prepared to analyze the relative viral genome entry by RT-PCR analysis. (**I**) CIK cells pre-treated with VP7 or endocytosis pathway inhibitor were infected with GCRV at 28°C for 1 h and prepared to quantify the relative viral genome entry by RT-PCR. Cells pre-treated with Vec as a control. (**J**) CIK cells pre-treated with VP7 in the presence or absence of AUY922 were infected with GCRV at 28°C for 1 h and prepared to quantify relative viral genome entry by RT-PCR. (**K**) CIK cells transfected with VP7 or vector plasmids were prepared for live cell imaging with Nikon confocal laser microscope system. (**L**) CIK cells treated with purified VP7 protein were prepared to analyze the morphological and structural change by electron microscopy analysis. (**M**) CIK cells pre-treated with VP7 were infected with GCRV at 28°C for 1 h and prepared to quantify the relative expression of many plasma membrane-related genes by RT-PCR. Data were presented as mean ± SD from three independent experiments. Statistical analysis was performed using one-way ANOVA between different groups and the asterisk (*) indicates significant differences between groups. * *p*<0.05, **p<0.01, ***p<0.001.

It has been previously reported that VP5 and VP7, the major outer capsid proteins of GCRV, are extremely important for establishing infection and have been well investigated as the potential vaccination target against GCRV infection [12]. We thus hypothesized that HSP90 may interact with them to facilitate GCRV entry. We first purified double-tagged fusion capsid proteins of GCRV, His-GFP-VP5, and His-GFP-VP7, by Ni-NTA-based affinity purification to homogeneity (Fig 5A and 5B). We then incubated these fusion proteins with GCO cells, microscopic analysis found that they bound to GCO cells (Fig 5C), indicating they are the immunogens of GCRV to mediate viral entry. To test whether VP5 and VP7 bind to HSP90 on the host cells to facilitate GCRV entry, we incubated tagged VP5 and VP7 with lysates of CIK cells and sent them for mass spectrometric analysis. Both samples from VP5 and VP7 identified HSP90 as their binding targets (S5A and S5B Fig), as well as cytoskeletal proteins (e.g., tubulin, actin, actinin), chaperone proteins (e.g., HSP70, HSP60), transport proteins (e.g., clathrin, dynein, kinesin, importin), and possible bona fide viral receptors (e.g., integrin β1, ephrin, fibronectin) (S4 and S5 Tables), suggesting that the early stage of GCRV infection involved tight crosstalk between viral antigen VP5/VP7, host molecular chaperons, receptors, and transport machinery, working together to facilitate the initial viral entry. To verify the interaction between VP5/VP7 and HSP90, we performed a pulldown assay using His-tagged VP5/VP7 fusion proteins. The data confirmed that both VP5 and VP7 interacted with HSP90, with a much stronger interaction between VP7 and HSP90 (S5C Fig), suggesting that VP7 plays an indispensable role in HSP90-mediated GCRV infection. Given that HSP90 is associated with STAT3 on the cellular membrane during temperature-switch treatment or GCRV infection, we examined whether VP7 interacted with HSP90 and STAT3 in CIK cells. Fractionation coupled with His pulldown analysis showed that, compared with VP5, VP7 was strongly associated with HSP90 and STAT3 in both the membrane and cytosolic fraction, where early endosome marker EEA1 resided (Fig 5D and 5E), suggesting that STAT3, HSP90, and VP7 may function as a complex facilitating viral entry. Further far-Western blotting analysis (a method derived from the standard Western blotting to detect protein-protein interaction *in vitro*) confirmed the direct interaction between VP7 and HSP90 on the PVDF membrane (Fig 5F), while HSP90 inhibitor 17-AAG treatment in CIK cells reduced the interaction between HSP90 and VP7 (S5D Fig). Furthermore, immunofluorescence analysis showed that VP7 colocalized with HSP90 to form aggregate or punctate signals in GCO, CIK, and 293T cells (Figs 5G and S5E). To probe the role of VP7 in GCRV infectivity, we found that pre-treated CIK cells with purified VP7 increased the viral entry of GCRV in a dose-dependent manner (Fig 5H), suggesting that VP7 pre-treatment could get the cells ready for viral entry. Conversely, with endocytosis inhibitors pre-treatment, such as ammonium chloride or nystatin nearly abolished the infection of GCRV (Fig 5I). Also, HSP90 inhibitor AUY922 treatment recapitulated the effect of endocytosis inhibition (Fig 5J), indicating that HSP90 is indispensable for VP7-mediated viral entry. To further probe the role of VP7 on host cells, we first performed a live cell imaging analysis of CIK cells stably expressing VP7 (Fig 5K and S1 and S2 Videos), we found that compared with the evenly distributed pattern of vector, VP7 displayed a more clustered pattern, which pulled the adjacent VP7 expressing cells together to form a bigger aggregate (Fig 5K and S2 Video), suggesting that VP7 plays important roles mediating viral spreads through cell-to-cell transmission. To further examine the subcellular structural change of host cells caused by VP7, we performed an electron microscopy analysis of CIK cells treated with purified VP7 protein, we found that CIK cells showed a typical fuzzy morphology on the plasma membrane when cells were treated with purified VP7 protein (Fig 5L), suggesting that the morphologic change on the plasma membrane may enable the landing and wrapping of virus particles during the initial infection stage. RT-PCR analysis further found that VP7 increased the expression of many genes involved in cell-to-cell communication and viral infection, including fibronectin, myosin, integrin, laminin receptor (LamR), and scavenger receptor class B type 1 (SRB1) (Fig 5M). As a control, we deployed another housekeeping gene GAPDH as the reference to confirm that VP7 or HSP90 did not influence the transcription of β-actin (S5F Fig). Collectively, these results supported the conclusion that VP7 interacts with HSP90 and STAT3 during GCRV infection, and promotes viral entry by inducing cytoskeletal rearrangement and expression of plasma membrane receptors, which ultimately get the cell ready for viral entry.

### IL6-STAT3-HSP90 axis mediates viral entry in aquatic ectotherms

**Fig 6:**
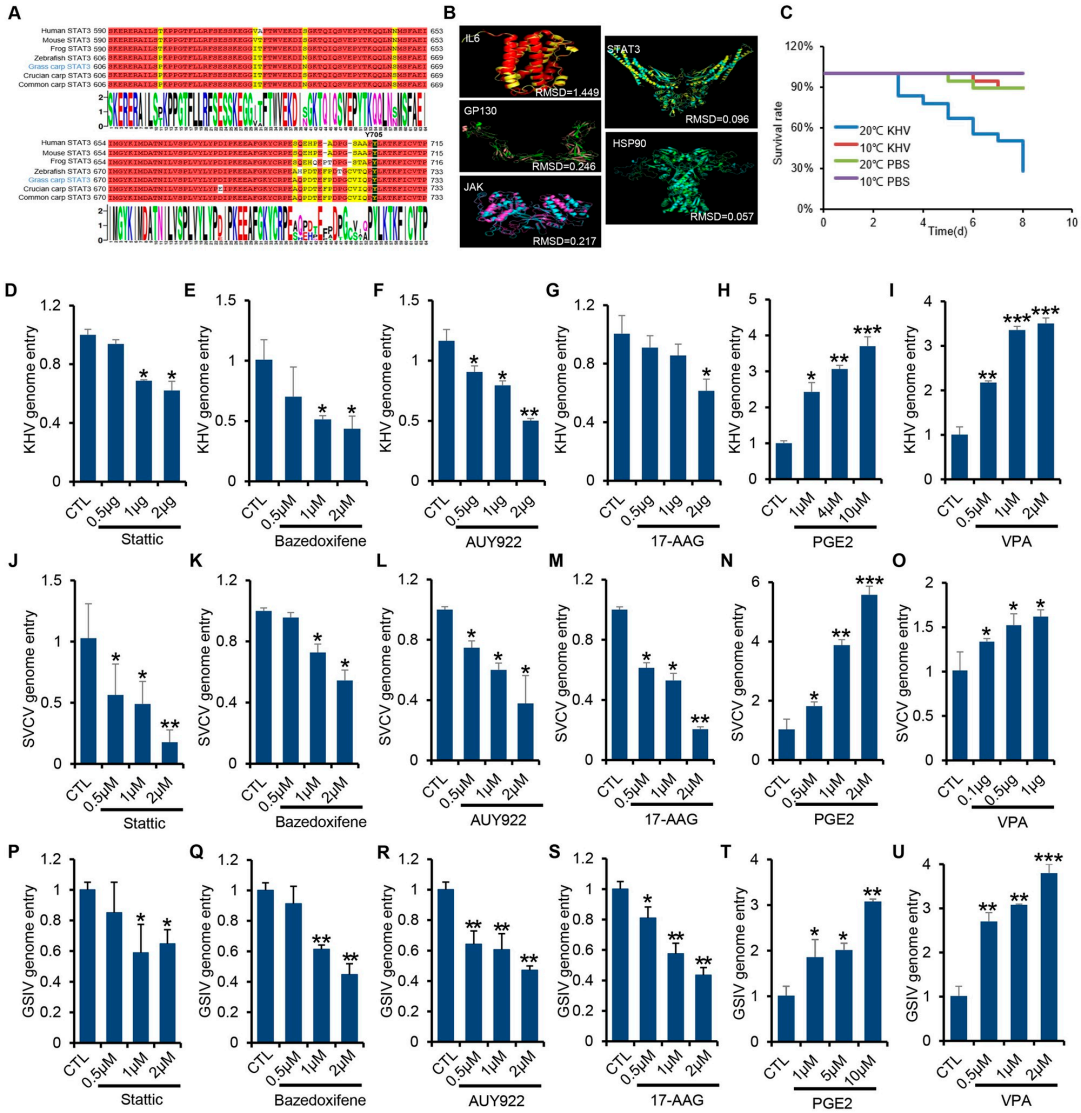
IL6-STAT3-HSP90 axis mediates viral entry in aquatic ectotherms. (**A**) Evolutionary conserveness analysis of STAT3 was conducted by multiple amino acids alignment of STAT3 from different species. The phosphorylation residue tyrosine 705 (Y705) which mediates the canonical STAT3 activation was highlighted in bold with dark shading. (**B**) Predicted three-dimensional structures of IL6-STAT3-HSP90 axis-related protein were performed with the I-TASSER program (https://zhanggroup.org/I-TASSER/) and visualized with the PyMOL program. (**C**) Koi (average weight 10 g) were intraperitoneally injected with KHV or PBS under different temperatures, and survival was monitored over 7 days. (**D-I**) CCB cells pre-treated with different doses of inhibitors or agonists (Stattic, bazedoxifene, AUY922, 17-AAG, PGE2, VPA) were infected with KHV (MOI = 5) at 22°C for 1 h and prepared to analyze the relative genome entry by RT-PCR with the ORF4L primer pair. (**J-O**) EPC cells pre-treated with different doses of inhibitors or agonists (Stattic, bazedoxifene, AUY922, 17-AAG, PGE2, VPA) were infected with SVCV (MOI = 5) at 22°C for 1 h and prepared to analyze the relative viral genome entry by RT-PCR analysis with the GP primer pair. (**P-U**) GSM cells pre-treated with different doses of inhibitors or agonists (Stattic, bazedoxifene, AUY922, 17-AAG, PGE2, VPA) were infected with GSIV (MOI = 5) at 22°C for 1 h and prepared to quantify the relative viral genome entry by RT-PCR with the MCP primer pair. Data were presented as mean ± SD from three independent experiments. Statistical analysis was performed using one-way ANOVA between different groups and the asterisk (*) indicates significant differences between groups. * *p*<0.05, **p<0.01, ***p<0.001.

To determine whether the mechanism of the IL6-STAT3-HSP90 axis regulating viral entry applies to other virus-host interactions in aquatic ectotherms, we first performed the amino acid alignment of IL6-STAT3-HSP90 axis-related proteins across species, including IL6, IL6R, gp130, JAK1, STAT3, and HSP90. We found that the sequence similarity was higher for downstream proteins within this axis (S3 Table). For example, the similarity score was over 85% in all kinds of vertebrates STAT3 and HSP90 (S3 Table). For STAT3, the key amino acid (Y705) that is required for the canonical phosphorylation-mediated activation of STAT3 was highly conserved through the evolution of vertebrates (Fig 6A). In addition, the predicted three-dimensional structures of IL6-STAT3-HSP90 axis related proteins in grass carp and humans by I-TASSER server [43] showed similar spatial structure, as shown by the pairwise model comparison analysis (Fig 6B), suggesting that the signaling transduction pathway is conserved during evolution. To explore whether the IL6-STAT3-HSP90 axis regulates viral infection in other aquatic ectotherms, we examined the koi herpesvirus (KHV) for koi and spring viremia of carp virus (SVCV, Rhabdovirus Carpio) for common carp. The optimal temperature for KHV and SVCV replication is around 22°C [44,45]. Survival rate analysis of KHV infection in koi showed a typical temperature-dependency, in which a higher temperature of infection (20°C) greatly increased the mortality of koi, while a lower temperature of infection (10°C) tended to preserve their survival (Fig 6C). RT-PCR analysis showed that lytic infection of KHV in common carp brain (CCB) cells (S6A Fig) or temperature-switch treatment of koi (10°C to 20°C) (S6B Fig) significantly induced the expression of IL6, suggesting that temperature-dependent IL6 expression may participate in the lytic infection of KHV. Furthermore, inhibitors of the IL6-STAT3-HSP90 axis, such as Stattic, bazedoxifene, AUY922, and 17-AAG, dampened the viral entry of KHV infection in a dose-dependent manner (Fig 6D–6G), while agonists of IL6-STAT3-HSP90 axis, such as PGE2 and VPA, increased the viral entry of KHV infection (Fig 6H and 6I). Meanwhile, the transcription of pro-inflammatory cytokines (IL6, TNF-α, IL1β) was likely parallel well with the inhibitors/agonist treatment (S6C–S6E Fig). In addition, the same was held with SVCV infection, we found that bazedoxifene/Stattic/17-AAG/AUY922/PGE2/VPA treatment inhibits or increases the SVCV genome entry, and the transcription of pro-inflammatory cytokines (IL6, TNF-α, IL1β) accordingly in a dose-dependent manner (Figs 6J–6O and S6F–S6H). Furthermore, we tested whether this mechanism can be extended to ectothermic amphibians. We employed the aforementioned inhibitors or activators to Chinese giant salamander muscle cells (GSM), then infected them with Chinese giant salamander iridovirus (GSIV). RT-PCR analysis showed that inhibition of the IL6-STAT3-HSP90 axis by inhibitor treatment (Stattic, bazedoxifene, 17-AAG, AUY922) diminished the viral entry (Fig 6P and 6S), while its activation by agonist treatment (PGE2, IL6, VPA) promoted the viral entry (Fig 6T and 6U). Cell viability assay showed that all the drugs we used under our experimental conditions had a negligible cytotoxic effect on the cells (S6I and S6J Fig). Altogether, these data suggest that the IL6-STAT3-HSP90 axis mediates viral entry in aquatic ectotherms.

## Discussions

**Fig 7:**
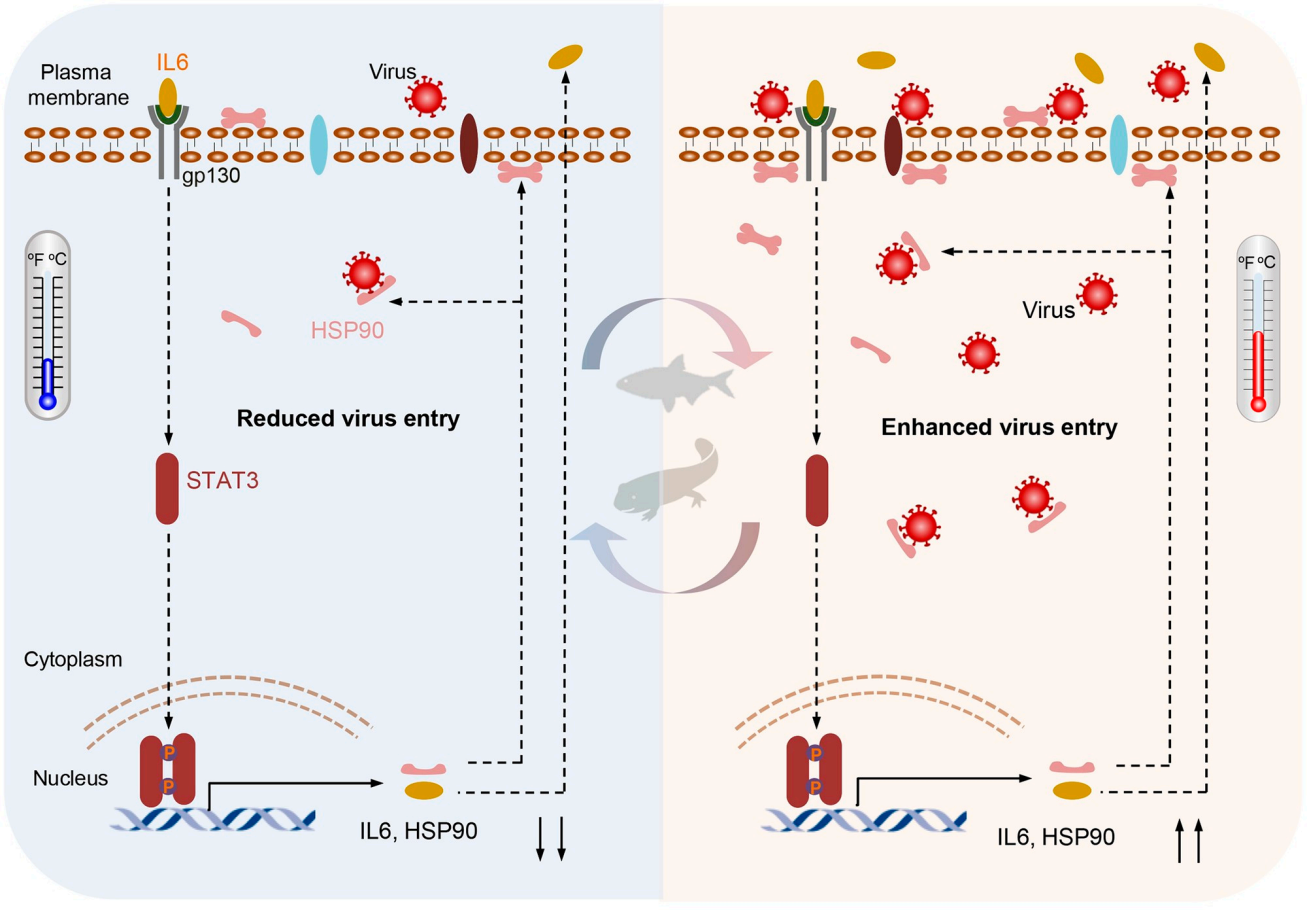
Graphical abstract. A model depicting the temperature-dependent activation of the IL6-STAT3-HSP90 signaling axis is exploited by aquatic viruses to facilitate viral entry into host cells. Temperature stress induces IL6-gp130 mediated P-STAT3 activation, which translocates into the nucleus to promote the transcription of pro-inflammatory cytokines (e.g., IL6, TNF-α) and heat-shock-related chaperone molecule HSP90. HSP90 is mainly positioned at the plasma membrane and is capable of bridging the interaction between viral proteins and host receptors to promote viral entry.

Viral diseases in the aquaculture industry show typical seasonality and temperature dependency phenotype which causes huge economic losses every year [9]. Here, utilizing GCRV infection in grass carp as a model to dissect the molecular mechanism behind the phenomenon, we identified that the IL6-STAT3-HSP90 axis played an important role in GCRV entry (Fig 7). We found that the temperature-switch evoked a quick immune response, induced the activation of IL6-STAT3 signaling pathway and the expression of HSP90. Interestingly, our data suggested that the subcellular localization of HSP90 is highly associated with the plasma membrane and its expression was regulated by IL6-STAT3 signaling (Fig 7). As a key chaperon protein downstream IL6-STAT3 pathway, HSP90 interacted with VP7, a major outer capsid protein of the GCRV, to induce a cytoskeletal rearrangement, morphological change, and induction of membrane-receptor related genes, which collectively advanced the penetration of the virus into host cells. Accordingly, exogenous expression of either IL6, HSP90, or VP7 in cells engendered an increased viral entry. Interestingly, we found that other aquatic viruses have evolved a similar mechanism to promote their infection. Taken together, we delineated for the first time the role of the IL6-STAT3-HSP90 axis exploited by aquatic viruses to benefit viral entry.

Virus-host-environment interaction is a theme that permeates the entire course of the coevolution of life, reflected not only by the arms race between viruses and their hosts but also by the cooperation whereby viruses contribute to cellular functions whereas cellular genes are picked up by viruses and employed for the essential viral functions such as replication or protein processing [46]. Taking the environment into account, in natural systems, it is quite common that viral infection rate and symptomatic diseases are highly season-dependent in aquaculture industries, from which viral diseases commonly prevail in summer while diminishing in winter [8]. However, little is known regarding how environmental temperature alters virus-host interactions. At this point, there are two ways to explain the phenomenon. The first is that both viral replication and host growth are highly temperature-dependent. Higher temperature tends to boost within-host virus replication and promote viral transmission, while lower temperature keeps within-host virus accumulation low, suggesting the importance of seasonal dynamics on within-host virus accumulation as a key determinant factor [47,48]. On the other hand, the seasonal dynamics on host responses, such as the antiviral immune defenses and the metabolic rate, could be another determinant factor contributing to the seasonality of viral diseases [48,49]. Yet, how seasonal temperature fluctuation impacts host responses and further shapes viral infection rates is still largely unknown. Our study utilized GCRV infection as a model to investigate the molecular mechanism of temperature dependency of aquatic viral diseases. The integrated data we provided strongly supports the hypothesis that temperature fluctuation might lead to imbalances of host innate immune responses, which further influence the within-host viral infection and transmission [48]. However, a more in-depth study of the seasonality in viral dynamics and virus-host interactions in naturally occurring infections needs to be evaluated in future studies, aiming to provide new insights into the multifaceted cooperation of virus-host-environment coevolution, particularly in the context of rapid global climate change in the past decades.

The growth factor- or cytokine-induced STAT3 activation plays pleiotropic roles in oncogenesis, host defense, and homeostatic maintenance [26,27]. Interestingly, both pro- and antiviral roles of STAT3 have been documented. These seemly contradictory responses appear to be dependent on the virus, the physiological status of the cells involved, and the activation of distinct sets of target genes transcriptionally regulated by STAT3 [10]. Notably, there is increasing evidence to suggest that STAT3 plays an important role in viral replication and pathogenesis, through a complex interplay between viruses and STAT3 signaling. For instance, STAT3 binds directly to the viral genome to promote viral gene expression, aberrant activation of STAT3 induces the expression of anti-apoptotic proteins, oncogenic proteins, and inflammation-related proteins, which are proposed to be beneficial to the replication of viruses and pathogenesis [4,10,50]. However, the precise role of STAT3 signaling in the early step of viral infection is not yet fully understood. To the best of our knowledge, our study is the first report showing that environmental temperature-dependent IL6-STAT3 signaling activation could mediate aquatic virus entry, which has allowed us to extend our understanding of the complex interplay between virus and IL6-STAT3 signaling. As the molecular mechanism, we found that HSP90, which is a ubiquitously expressed chaperon protein that contributes to maintaining the proper folding, maturation, trafficking, stability, and activity of numerous client proteins [51], by itself is closely associated with STAT3, and can be regulated by IL6-STAT3-signaling. Our data suggested that HSP90 is a pivotal chaperon protein for viral infection, and may function as a co-receptor for virus entry through binding directly with the outer capsid proteins of GCRV. Our finding is consistent with the reported study demonstrated that HSP90 localized at the plasma membrane and was essential for GCRV-II entry via the clathrin-mediated endocytosis pathway [21]. Plenty of laboratory studies have investigated that the IL6-STAT3 pathway is an inducer of HSP90 [27,29,52], and HSP90 is usually exploited by a broad range of viruses to function as a co-receptor or key scaffold to act as an enhancer of viral entry [37,39,53]. Meanwhile, the notion that HSP90 may serve as a key molecule to mediate various viral entries raised several questions to be addressed. Given the broad range of viruses and viral client proteins HSP90 may target in general, the molecular mechanism in detail is far from clear. Whether a general mechanism of HSP90 mediating viral entry exists? Whether the ATPase activity of HSP90 is required to assist the process? Whether HSP90 promotes viral entry by converting subtle changes in the conformation of bona fide viral receptors to switch their ligand binding affinity or inducing and maintaining the post-translational modification of viral receptors for activation, resembling the examples in which HSP90 activates steroid hormone receptor and protein kinase cyclin-dependent kinase Cdk4 by inducing clients conformational change and phosphorylation, respectively [54]. Besides, our data suggested the primary plasma and endosomal membrane-associated HSP90 might form a complex with VP7 and STAT3 to mediate GCRV entry into host cells, which appears to conflict with other studies reporting that HSP90 is primarily cytoplasmic or secreted [55]. This discrepancy could be attributed to the cell type or differences in methodologies. A more in-depth mechanistic study involving HSP90-mediated viral entry needs to be conducted to address these unanswered questions in the future. Also, we do not exclude the possibility that other HSPs or cell membrane receptors participate in this process. As our transcriptomic and RT-PCR data from STAT3-deficient zebrafish and Stattic treated cells showed that STAT3 also regulates many genes expression involving cell adhesion, cytoskeleton, and HSPs. Lastly, how does the IL6-STAT3 axis transmit the signal to HSP90 to facilitate viral entry during the early stages of viral infection? Is STAT3 involved in any non-transcriptional actions that contribute to viral entry, other from its role as a transcription factor in regulating HSP90 production? Further in-depth studies to dissect the precise role of STAT3 in mediating viral early infections could provide more valuable insights into viral pathogenesis. In addition, the activation of IL6-STAT3 signaling and HSP90 expression can be impacted not only by temperature fluctuation, but also by other environmental factors/stimuli during the farming process of aquatic animals, such as ammonia nitrogen, dissolved oxygen, salinity, pH, and stocking density [56,57]. Thus, our study suggests that induced expression of HSP90 by environmental stimuli conditions may increase the susceptibility to viral infection, which raises intriguing questions about the relationship between HSP90 expression, virus infection, and host health in aquaculture industry. As emerging investigations showed that manipulated expression of HSPs through either feed addictive supplementation, genetic selection, housing management, or vaccine implementation could confer a cytoprotective role for animal heat resistance in livestock and aquaculture industry [58,59]. The roles of HSPs for host health and diseases appear counterintuitive depending on the context and host susceptibility. However, our understanding concerning how the environmental stress responses in livestock and aquaculture industry alter the interaction between pathogens and host immune system, as well as the further development of infectious diseases is quite incomplete. Nevertheless, our data uncovered a typical immune evasion strategy exploited by the viruses to benefit viral entry during early infection, providing critical mechanistic insight into the temperature-dependent viral pathogenesis.

Temperature dependency of viral diseases in the aquaculture industry is a prevailing phenomenon, which causes enormous economic losses worldwide every year [9,60]. Our work suggested the molecular mechanism that temperature-related IL6-STAT3-HSP90 signaling mediates GCRV entry, might be universal in aquatic ectotherms, pinpointing an opportunity to develop innovative preventives to treat such diseases in the aquaculture industry thereof. Furthermore, from an evolutionary point of view, on account of the pro-survival roles IL6/STAT3/HSP90 endow, whether such a mechanism we identified in aquaculture is universally conserved in the interplay between virus and host coevolution from ectotherms to endotherms is an intriguing question for future studies. Nevertheless, our work provides further credence to the pivotal roles of IL6 and HSP90 as risk factors of susceptibility to viral infection, highlighting the feasibility and rationality of developing targeted preventives towards IL6-STAT3 signaling and HSP90 for viral diseases in the future.

## Materials and methods

### Animal ethic statements

All procedures with grass carp, koi fish, and zebrafish used in experiments were approved by the Ethics Committee of Hunan Agriculture University and the methods were carried out following the approved guidelines of animal ethics statements.

### Fish

Grass carp about 8–10 cm in length and koi fish about 6–8 cm in length were utilized for viral infection or temperature-switch experiments. Adult zebrafish were raised and kept in a recirculating system at 28°C with a 14 h light/h dark cycle.

### Cells

Fish cell lines such as CIK (*Ctenopharyngodon idellus* kidney cells), GCO (Grass carp ovary cells), CCB (Common carp brain cells), EPC (*Epithelioma Papulosum Cyprini* cells), GSM (Chinese giant salamander muscle cells) were maintained in medium 199 (M199) supplemented with 10% fetal bovine serum (FBS), penicillin (100 U/ml) and streptomycin (100 μg/ml) at 28°C in carbon dioxide-free atmosphere. HEK293T (human embryonic kidney cells) were maintained in Dulbecco’s modified Eagle’s medium (DMEM) supplemented with 10% FBS, penicillin (100 U/ml), and streptomycin (100 μg/ml) at 37°C in a humidified 5% CO_2_ atmosphere.

## Method details

### Virus preparation

GCRV-JX01 (GCRV-I strain) and GCRV-AH528 (GCRV-II strain) were deployed to infect CIK and grass carp, respectively. GCRV-JX01, koi herpesvirus (KHV), spring viremia of carp virus (SVCV), and Chinese giant salamander iridovirus (GSIV) were amplified in CIK, CCB, EPC, and GSM cells accordingly. The supernatant containing infectious virion particles was collected and centrifuged at 1000 g for 10 min. The virus was concentrated by ultracentrifugation at 32,500 g for 2 h, if necessary.

### Temperature-switch experiment

Grass carp about 8–10 cm in length were temporarily raised in a tank of 18°C water temperature for 1 week of adaptation. After then the fish were transferred to a tank at 28°C water temperature. Tissue samples were collected at different time points for RT-PCR analysis. CIK or GCO cells were cultured at 18°C or 28°C cell incubators for 48 h for adaptation, GCRV infection and temperature stress switching experiments were then conducted from 28°C to 18°C or 18°C to 28°C, respectively. Cell samples at different time points were collected to analyze the relative gene expression and viral genome replication. Temperature-switch experiment for koi was conducted accordingly.

### DNA transfection

All the plasmid transfections in cells were performed using polyethylenimine (PEI) following the manufacturer’s protocol. Cells were seeded into 6-well plates to the density of 50–70% confluency. Serum media were removed from cells and replaced with serum-free DMEM media. Dilute plasmid DNA (2 μg) and 6 μL PEI (1 mg/mL) into a 100 μL OPTI medium separately. After incubation for 3 min, the PEI solution was mixed into the DNA solution and incubated at room temperature for 15–20 min. The transfection mixture was added slowly to the cells (200 μL per well) and incubated for 4 h. After then, Media were replaced with complete DMEM and incubated cells for 24 h-48 h based on the experimental requirements.

### siRNA assay

Four siRNAs targeting gcSTAT3 and three siRNAs targeting HSP90 were designed by Sangon Biotech Company (Shanghai, China). A control siRNA (NC) that has no homology with gcSTAT3 or gcHSP90 mRNA was used as a control. CIK cells were transfected separately with gcSTAT3 siRNA, gcHSP90 siRNA or NC using Invigentech INVI DNA RNA according to the instructions of the manufacturer for 24 h. After then the cells were infected with GCRV at 28°C for 1 h for virus entry quantification and 8 h for virus proliferation quantification, respectively, by RT-PCR analysis.

### Quantitative Real-Time PCR (RT-PCR)

Total RNA was extracted using RNA-easy Isolation Reagent (Vazyme), followed by cDNA preparation by the ReverAid First Strand cDNA Synthesis Kit (Thermo Scientific) according to the manufacturer’s instructions. RT-PCR reaction was performed with SYBR Master Mix (Vazyme). Relative mRNA expression for each target gene was calculated by the 2^−ΔΔCt^ method using β-actin as an internal control. Sequences of RT-PCR primers are listed in S2 Table.

### Inhibitor or activator assay

Cells were pretreated with different doses of inhibitors or activators dissolved in H_2_O or 10% DMSO for 2 h, in which Stattic was STAT3 inhibitor, AUY922 and 17-AAG were Hsp90 inhibitors, Bazedoxifene was gp130 inhibitor, IL6, valproic acid (VPA) and Prostaglandin E2 (PGE2) were IL6-STAT3 pathway activators. Infect cells with indicated viruses for different time points to examine either viral genome entry or replication. Total RNA was extracted for RT-PCR or transcriptomic sequencing analysis (Novegene).

### Transcriptomic analysis

Transcriptomic sequencing analysis was performed by the Novegene company. Briefly, total RNA was extracted using RNA-easy Isolation Reagent (Vazyme), followed by the RNA integrity assessment using the RNA Nano 6000 Assay Kit (Agilent Technologies, CA, USA). Qualified RNA was subjected to library preparation for transcriptomic sequencing. The clustering of the index-coded samples was performed on a cBot Cluster Generation System (Illumia). After cluster generation, the library preparations were sequenced on an Illumina Novaseq platform and 150 bp paired-end reads were generated. Differential expression analysis of two conditions/groups (three biological replicates per condition) was performed using the DESeq2 R package (1.20.0), *P*-value < = 0.05. KEGG enrichment analysis of differentially expressed genes was implemented by the cluster Profiler R package, in which gene length bias was corrected, *P*-value < = 0.05.

### Protein expression and purification

pCold-TF-EGFP-VP7 or pCold-TF-EGFP-VP5 plasmid was transformed into *E*. *coli* BL21(ED3). Recombinant His-VP5 or His-VP7 expression was induced by 0.1 mM IPTG at 16°C overnight. Bacteria were harvested, lysed, and sonicated with lysis buffer (containing 10 mM imidazole and protease inhibitor, pH 8.0). The supernatant was incubated with Ni-beads (Beyotime) for 2 h at 4°C. Precipitated beads were washed 10–15 times with wash buffer (contain 40 mM imidazole, pH 8.0) and resolved by SDS-PAGE analysis. Samples were then subjected to perform pulldown, immunoblotting, or **mass spectrometry analysis**.

### Western blot and Far-Western blot

Cells were harvested and lysed with RIPA lysis buffer (Beyotime) supplemented with protease inhibitor cocktail. Whole cell lysates were sonicated and centrifuged at 12000 rpm at 4°C for 30 min. The supernatant was resolved with 5×SDS loading buffer by boiling at 98°C for 10 min. Samples were then separated by SDS-PAGE and followed by transferring to PVDF membrane. Transfer efficiency was checked by Ponceau S staining before probing the primary antibody. After that, the membranes were blocked with 4% skimmed milk in TBST for 1 h at room temperature. All immunoblotting analyses were performed using indicated primary antibodies (1:1000–1:2000 dilution in TBST) for 2 h at room temperature and HRP-conjugated secondary antibody (1:2000–1:4000 dilution in TBST) for 1 h at room temperature. Proteins were visualized using an ECL detection kit (Beyotime). For far-Western blot, cells were prepared as the western blot procedure described. After the PVDF membranes were blocked with 4% skimmed milk, the membranes were incubated with the purified recombinant protein His-VP7 overnight at 4°C. The membranes were then subjected to probe with primary anti-His antibody and HRP-conjugated secondary antibody, followed by signal visualization by an ECL detection kit.

### CO-IP and his pulldown assays

Cells were harvested and lysed with NP40 buffer (50 mM Tris-HCl pH 8.5, 150 mM NaCl, 1% NP40) supplemented with a protease inhibitor cocktail. WCLs were sonicated for 20 min in ice and centrifugated at 12000 rpm at 4°C for 30 min. The supernatant was precleared with protein A/G agarose beads at 4°C for 1 h and then incubated with the corresponding antibody at 4°C overnight. Agarose beads were washed with lysis buffer and eluted with 1× SDS loading buffer by boiling at 98°C for 10 min. Samples were then subjected to SDS-PAGE and immunoblotting analysis.

For His pulldown assay, CIK cells were lysed in NP40 buffer supplemented with a protease inhibitor cocktail. Cell lysates were centrifugated and precleared with Ni beads. Precleared cell lysates were incubated with purified recombinant protein His-VP7 or His-VP5. After incubation at 4°C for 4–6 h, the resin was washed three times with NP40 buffer and then resolved by SDS-PAGE-based immunoblotting or mass spectrometry analysis.

### Immunofluorescence microscopy and live cell imaging

Cells were processed as previously described with some modifications [61]. Briefly, cells expressing fluorescent fusion GFP or RFP were fixed with 4% paraformaldehyde and stained with 4, 6-diamidino-2-phenylindole (DAPI). After that, cells were directly analyzed by an immunofluorescence microscope (Nikon). For live cell imaging, CIK cells were seeded on a chambered cover glass at a density of around 50% confluency. Cells were then transfected with corresponding plasmids and videos were recorded using Nikon confocal laser microscope system.

### Subcellular fractionation

CIK cells (2 × 10^6^) were harvested and washed with 3 mL of cell wash solution. The cell pellet was suspended with 0.75 mL permeabilization buffer to incubate for 10 min at 4°C with constant mixing. Centrifuge permeabilized cells for 15 min at 16,000 × *g* to get the supernatant containing cytosolic protein fractions. 0.5 mL of solubilization buffer was then added to the pellet and incubated at 4°C for 30 min with constant mixing. Membrane and membrane-associated fractions were obtained by centrifuging the solubilization solution at 16,000 × *g* for 15 min at 4°C.

### STAT3 mutant zebrafish generation

STAT3-KO mutant zebrafish were generated by TALEN technology as previously described [62]. Briefly, the TALEN right and left arms for STAT3 were designed by the TALE-NT program and synthesized using the TALEN kit according to the manufacturer’s protocol. The linearized TALEN plasmids were transcribed into mRNAs using the T7 mMessage mMachine kit. The synthesized mRNAs were purified with lithium chloride precipitation and injected at a dose of 300 pg into the zebrafish embryo at the one-cell stage. Genotyping PCR was conducted to determine the mutant zebrafish. The mutated STAT3 codes for a truncated protein containing 165 aa, 46 aa of which at the N-terminal is identical to wildtype STAT3.

### Survival assay

The experimental koi were kept for a week at 10°C and were divided into two groups: the koi fish were intraperitoneally injected with KHV at 20°C (n = 30) and 10°C (n = 30). the control group was injected with PBS at 20°C (n = 30) and 10°C (n = 30). The survival rate was calculated by counting the number of dead fish every day.

### Mass spectrometry (MS) analysis

Recombinant protein His-VP5 or His-VP7 was purified from *E*. *coli* BL21(ED3) and transformed with plasmid pCold-TF-EGFP-VP5 or pCold-TF-EGFP-VP7 by affinity chromatography using Ni-beads. Purified His-VP5 or His-VP7 was then incubated with CIK whole cell lysate. The incubated beads were washed three times with PBS, followed by adding a reaction solution containing SDC, TCEP, and CAA for one-step reduction, alkylation, and elution, respectively. After that, the samples were subjected to enzymatic hydrolysis by trypsin and mass spectrometry analysis using Q EXactive Plus liquid mass spectrometry system (Thermo). The samples were separated by a liquid phase UltiMate 3000 RSLCnano system, with a flow rate being 300 nL per minute.

### Electron microscopy

Electron microscope observation of CIK cells infected with GCRV or treated with purified VP7 was performed as previously described [63]. In brief, CIK cells were fixed by 2.5% glutaraldehyde overnight at 4°C. Then the cells were post-fixed in 1% osmium tetroxide for 1 h, followed by washing, dehydration, embedding, and sectioning steps. After that, the cells were stained using 2% uranyl acetate and lead citrate. Images were acquired by transmission electron microscopy (Hitachi-7650, Tokyo, Japan).

### Statistical analysis

Statistical analysis was performed using unpaired two-tailed Student’s t-test or one-way ANOVA between different groups. A *P*-value <0.05 was considered statistically significant. *P<0.05, ** *P*<0.01, ***P<0.001. Data are represented by three independent experiments and shown as the mean ± SD. For heatmap analysis, the transcriptomic data matrix normalized by autoscaling was exported into a txt file and analyzed using TBtools software interface with an in-house R script.

## Supporting information

S1 Fig Temperature dependency of GCRV infection. (**A**) Grass carp hemorrhagic disease survey from 46 fisheries distributed in Yiyang and Changsha city in Hunan province were investigated. The original shp format file data for the map was obtained from the National Basic Geographic Information System (https://www.tianditu.gov.cn/) under license CC by 4.0 and then edited with ARCGIS software. (**B**) Hemorrhagic symptoms were observed in gills, fins, skins, and muscles in Fig 1B. (**C**) Grass carp were intraperitoneally infected with GCRV-AH528 (100 μL at 1.0 ×10^6^ TCID_50_ mL^-1^ per fish) at different temperatures, and the relative GCRV genome level from gill and intestine by day 5 post-infection was analyzed by RT-PCR with the VP4 primer pair. (**D**) The relative GCRV genome replication from infected GCO cells (Multiplicity of infection, MOI = 5) under different temperatures was analyzed by RT-PCR with the VP7 primer pair. Data were presented as mean ± SD from three independent experiments. Statistical analysis was performed using one-way ANOVA between different groups and the asterisk (*) indicates significant differences between groups. ***p<0.001.
(TIF)

S2 Fig IL6-STAT3 signaling pathway mediates temperature-dependent GCRV infection. (**A**) Heat map analysis of DEGs that were up-regulated by GCRV infection in the GCRV-infected head kidney tissue was summarized (NCBI SRA database accession number PRJNA759556). (**B**) Gills from Figs 1C and S1C were collected to analyze the relative expression of proinflammatory genes (IL6, IL1β, TNF-α). (**C-D**) Gills (C) and intestines (D) of grass carp under temperature-switch treatment from 18°C to 28°C were prepared to analyze the relative expression of proinflammatory genes (IL6, IL1β, TNF-α) by RT-PCR. (**E-G**) CIK cells infected with GCRV (MOI = 5) under different temperatures were prepared to quantify the relative expression of IL1β, TNF-α, gp130, IL6R, integrin-α4, JAMA, and HIF1α. (**H**) CIK cells pre-treated with different doses of Stattic were infected with GCRV (MOI = 5) at 28°C and harvested to analyze the expression of IL1β and TNF-α by RT-PCR. (**I**) CIK cells were treated with different doses of Stattic for 2 hours and harvested to probe the signals of STAT3 by Western blotting analysis. The density of the Western blot bands was quantified using ImageJ software. (**J**) CIK cells treated with different doses of Stattic were prepared to analyze the cell viability by trypan blue staining. zinc diethyldithiocarbamate (ZDEC) served as a positive control. Data were presented as mean ± SD from three independent experiments. Statistical analysis was performed using one-way ANOVA between different groups and the asterisk (*) indicates significant differences between groups. *p<0.05, **p<0.01, ***p<0.001.
(TIF)

S3 Fig IL6-STAT3-HSP90 signaling axis mediates temperature dependency of GCRV infection. (**A**) CIK cells transfected with different doses of STAT3-C plasmids were prepared to quantify the relative expression of IL6, IL1β, and TNF-α by RT-PCR analysis. (**B**) CIK cells transfected with different doses of STAT3-C plasmids were infected with GCRV (MOI = 5) for 8 hours and prepared to quantify the relative GCRV genome entry by RT-PCR analysis. (**C**) 293T cells transfected with different doses of STAT3-C plasmids were prepared to quantify the relative expression of IL6 and HSP90 by RT-PCR analysis. (D-F) CIK cells transfected with four different STAT3 siRNAs were infected with GCRV (MOI = 5) for 8 hours and prepared to quantify the transcription of IL6, IL1β, and TNF-α, and HSP90 (D) and the relative GCRV genome entry (E) by RT-PCR analysis. The STAT3 knockdown efficiency was analyzed by RT-PCR (F) and Western blotting (G). (**H**) CIK cells transfected with pEGFP-HSP90 were prepared to perform fluorescence analysis. pEGFP-N1 served as a control. (**I-J**) Gills of grass carp (I) or CIK cells (J) under temperature-switch treatment from 18°C to 28°C were prepared to quantify the relative expression of HSP90 by RT-PCR. Data were presented as mean ± SD from three independent experiments. Statistical analysis was performed using one-way ANOVA between different groups and the asterisk (*) indicates significant differences between groups. *p<0.05, **p<0.01, ***p<0.001.
(TIF)

S4 Fig IL6-STAT3-HSP90 signaling axis regulates GCRV entry. (**A**) Relevant activators or inhibitors involved in the IL6-STAT3-HSP90 signaling axis were utilized in this study. (**B**) CIK cells treated with different doses of drugs were prepared to analyze the cell viability by trypan blue staining. ZDEC served as a positive control. (**C**) CIK cells pre-treated with different doses of PGE2 were prepared to analyze the relative transcription of IL6 by RT-PCR. (**D**) CIK cells transfected with different doses of grass carp IL6 plasmids were prepared to quantify the relative transcription of IL6 by RT-PCR. (**E-F**) CIK cells transfected with different doses of HSP90 plasmids were infected with GCRV (MOI = 5) at 28°C for 1 h and harvested to quantify the relative transcription of HSP90, integrin α4, JAMA, and LamR by RT-PCR. (**G**) CIK cells treated with AUY922, 17-AAG, or bazedoxifene were infected with GCRV at 28°C for 8 h and harvested to quantify the relative viral genome replication by RT-PCR. (**H**) CIK cells from S4G Fig were prepared for CPE monitoring by optical microscope analysis. (**I**) CIK cells transfected with three different HSP90 siRNAs were infected with GCRV (MOI = 5) for 1 hour and prepared to quantify the relative transcription and protein translation of HSP90. Data were presented as mean ± SD from three independent experiments. Statistical analysis was performed using one-way ANOVA between different groups and the asterisk (*) indicates significant differences between groups. *p<0.05, **p<0.01, ***p<0.001.
(TIF)

S5 Fig HSP90 interacts with VP7 and STAT3 on the cellular membrane to promote GCRV entry. (**A-B**) CIK cell lysates were incubated with VP5-HIS-EGFP coupled with Ni-beads, and the pulldown complex was then prepared for mass spectrometry analysis. The mass/charge ratio (m/z) spectrum peptide analysis identified HSP90 as a binding candidate of VP7 (A) and VP5 (B). (**C**) CIK cell lysates were incubated with purified his-tagged VP5/VP7 protein-coupled by Ni-beads, and the precipitates from the pulldown complex were prepared to confirm the interaction between VP5/VP7 and endogenous HSP90 in CIK cells. (**D**) CIK cells treated with 17-AAG were prepared to examine the interaction between purified his-tagged VP7 and endogenous HSP90 from CIK cell lysates by pulldown analysis. (**E**) GCO, CIK, or 293T cells stably expressing tagged pEGFP-N1 and pDsRed were prepared to perform immunofluorescence analysis. Scale bars: 20 μm. (**F**) CIK cells transfected withdifferent doses of HSP90 or VP7 plasmids were prepared to quantify the relative transcription of β-actin with GAPDH as the reference gene.
(TIF)

S6 Fig IL6-STAT3-HSP90 axis mediates viral entry in aquatic ectotherms. (**A**) CCB cells under lytic infection of KHV at 22°C were prepared to quantify the relative transcription of an early gene of KHV ORF1L and IL6 by RT-PCR. (**B**) Gills from Koi (average weight 10 g) under temperature-switch treatment from 10°C to 20°C for different time points were harvested to analyze the relative expression of IL6 by RT-PCR. (**C-E**) CCB cells were treated with different doses of Stattic (C), bazedoxifene (D), or PGE2 (E) for 2 hours, followed by KHV infection for 1 hour. The cells were then harvested to quantify the relative transcription of IL6, TNF-α, and IL1β by RT-PCR. (**F-H**) EPC cells were treated with different doses of Stattic (C), bazedoxifene (D), or PGE2 (E) for 2 hours, followed by SVCV infection for 1 hour. The cells were then harvested to quantify the relative transcription of IL6, TNF-α, and IL1β by RT-PCR. **(I-J)** EPC or GSM cells pre-treated with different doses of drugs for 2 hours were prepared to analyze the cell viability by trypan blue staining. ZDEC served as a positive control. Data were presented as mean ± SD from three independent experiments. Statistical analysis was performed using one-way ANOVA between different groups and the asterisk (*) indicates significant differences between groups. *p<0.05, **p<0.01, ***p<0.001.
(TIF)

S1 Video CIK cells transiently transfected with pSDred-N1 vector plasmid were recorded using Nikon confocal laser microscope system. (AVI)

S2 Video CIK cells transiently transfected with pSDred-N1-VP7 plasmid were recorded using Nikon confocal laser microscope system. (AVI)

S1 Table Reagent and resource. (XLSX)

S2 Table Primers in this study. (XLSX)

S3 Table Amino acid similarity of IL6-STAT3-HSP90 axis related proteins between species. (XLSX)

S4 Table Proteins list summary identified by VP5 pulldown mass spectrometry analysis. (XLSX)

S5 Table Proteins list summary identified by VP7 pulldown mass spectrometry analysis. (XLSX)
