# Correction: An aquatic virus exploits the IL6-STAT3-HSP90 signaling axis to promote viral entry

**DOI:** 10.1371/journal.ppat.1011444

**Published:** 2023-06-12

**Authors:** Guoli Hou, Zhao Lv, Wenzhi Liu, Shuting Xiong, Qiushi Zhang, Chun Li, Xiaodong Wang, Liang Hu, Chunhua Ding, Rui Song, Hongquan Wang, Yong-An Zhang, Tiaoyi Xiao, Junhua Li

In the Funding section, the grant numbers from the funder Natural Science Foundation of Hunan Province of China are listed incorrectly. The correct grant numbers are: 2021JJ30326 and 2021JJ30336.
