# Supplementary figures and images for: An aquatic virus exploits the IL6-STAT3-HSP90 signaling axis to promote viral entry
Source: PLoS Pathog. 2023 Apr 26;19(4):e1011320. doi: 10.1371/journal.ppat.1011320 (PMC10166480; doi:10.1371/journal.ppat.1011320)

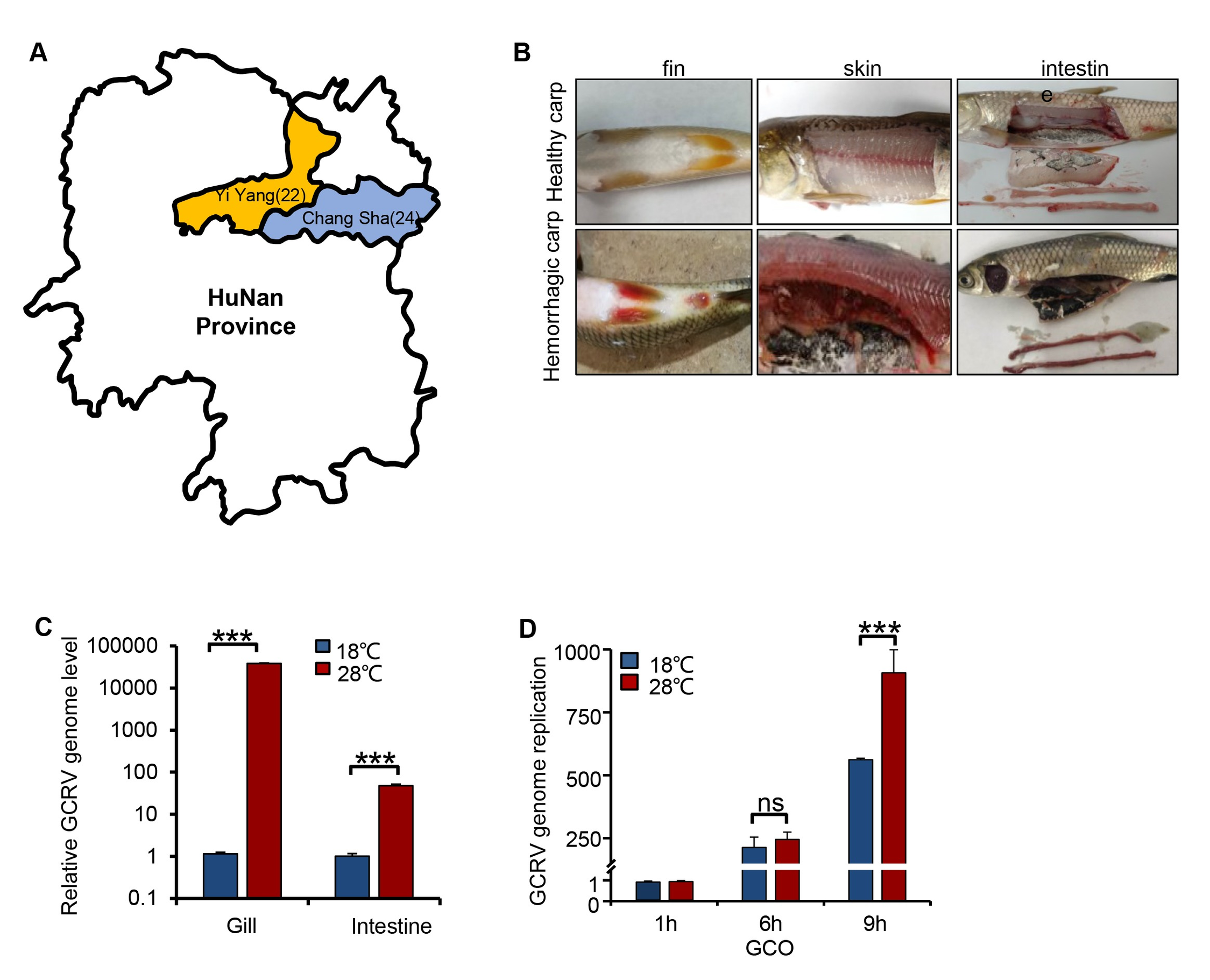

Supplement: S1 Fig — (A) Grass carp hemorrhagic disease survey from 46 fisheries distributed in Yiyang and Changsha city in Hunan province were investigated. The original shp format file data for the map was obtained from the National Basic Geographic Information System (https://www.tianditu.gov.cn/) under license CC by 4.0 and then edited with ARCGIS software. (B) Hemorrhagic symptoms were observed in gills, fins, skins, and muscles in Fig 1B. (C) Grass carp were intraperitoneally infected with GCRV-AH528 (100 μL at 1.0 ×10^6^ TCID50 mL-1 per fish) at different temperatures, and the relative GCRV genome level from gill and intestine by day 5 post-infection was analyzed by RT-PCR with the VP4 primer pair. (D) The relative GCRV genome replication from infected GCO cells (Multiplicity of infection, MOI = 5) under different temperatures was analyzed by RT-PCR with the VP7 primer pair. Data were presented as mean ± SD from three independent experiments. Statistical analysis was performed using one-way ANOVA between different groups and the asterisk (*) indicates significant differences between groups. ***p<0.001. (TIF) [file ppat.1011320.s001.tif]

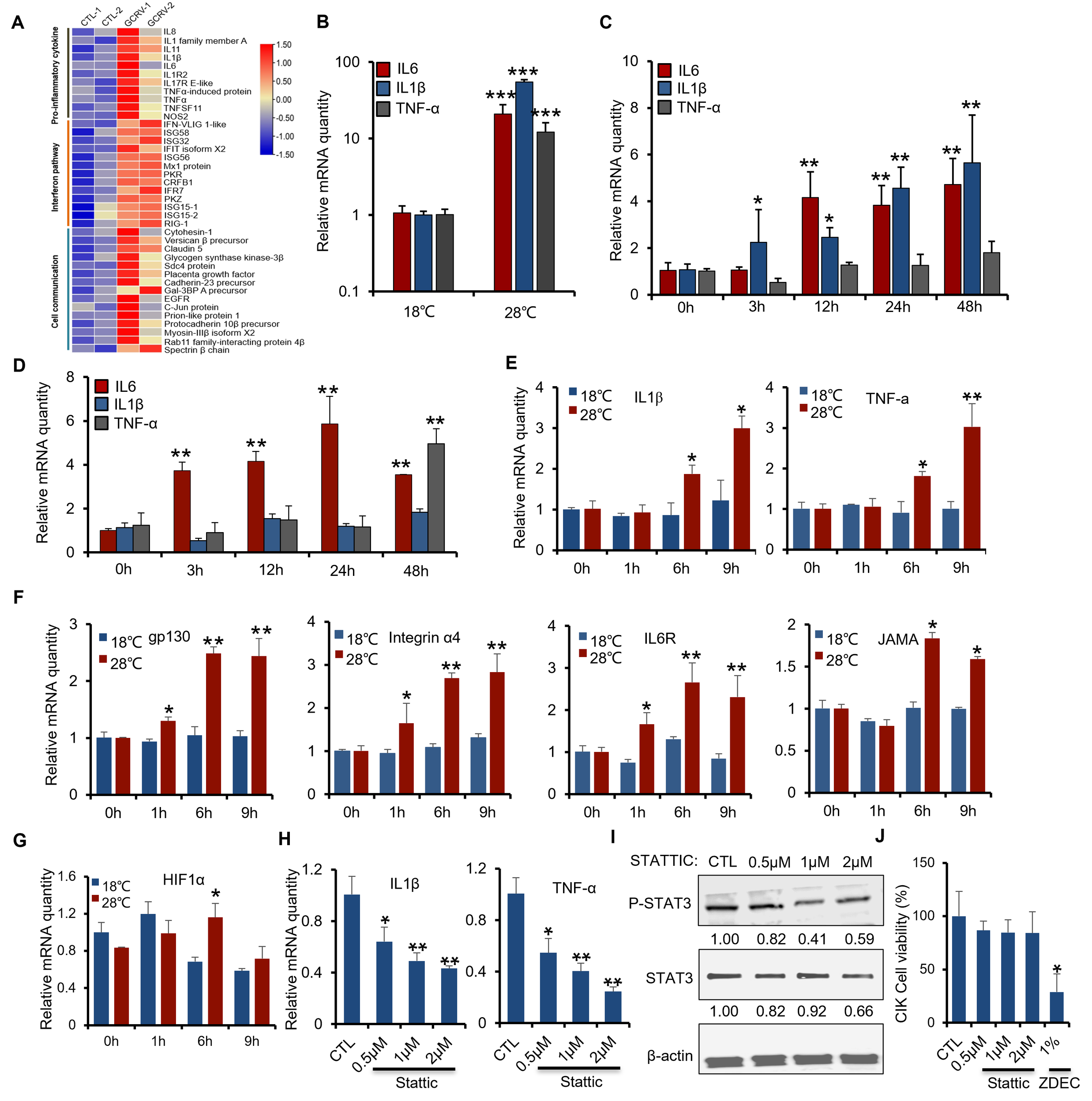

Supplement: S2 Fig — (A) Heat map analysis of DEGs that were up-regulated by GCRV infection in the GCRV-infected head kidney tissue was summarized (NCBI SRA database accession number PRJNA759556). (B) Gills from Figs 1C and S1C were collected to analyze the relative expression of proinflammatory genes (IL6, IL1β, TNF-α). (C-D) Gills (C) and intestines (D) of grass carp under temperature-switch treatment from 18°C to 28°C were prepared to analyze the relative expression of proinflammatory genes (IL6, IL1β, TNF-α) by RT-PCR. (E-G) CIK cells infected with GCRV (MOI = 5) under different temperatures were prepared to quantify the relative expression of IL1β, TNF-α, gp130, IL6R, integrin-α4, JAMA, and HIF1α. (H) CIK cells pre-treated with different doses of Stattic were infected with GCRV (MOI = 5) at 28°C and harvested to analyze the expression of IL1β and TNF-α by RT-PCR. (I) CIK cells were treated with different doses of Stattic for 2 hours and harvested to probe the signals of STAT3 by Western blotting analysis. The density of the Western blot bands was quantified using ImageJ software. (J) CIK cells treated with different doses of Stattic were prepared to analyze the cell viability by trypan blue staining. zinc diethyldithiocarbamate (ZDEC) served as a positive control. Data were presented as mean ± SD from three independent experiments. Statistical analysis was performed using one-way ANOVA between different groups and the asterisk (*) indicates significant differences between groups. *p<0.05, **p<0.01, ***p<0.001. (TIF) [file ppat.1011320.s002.tif]

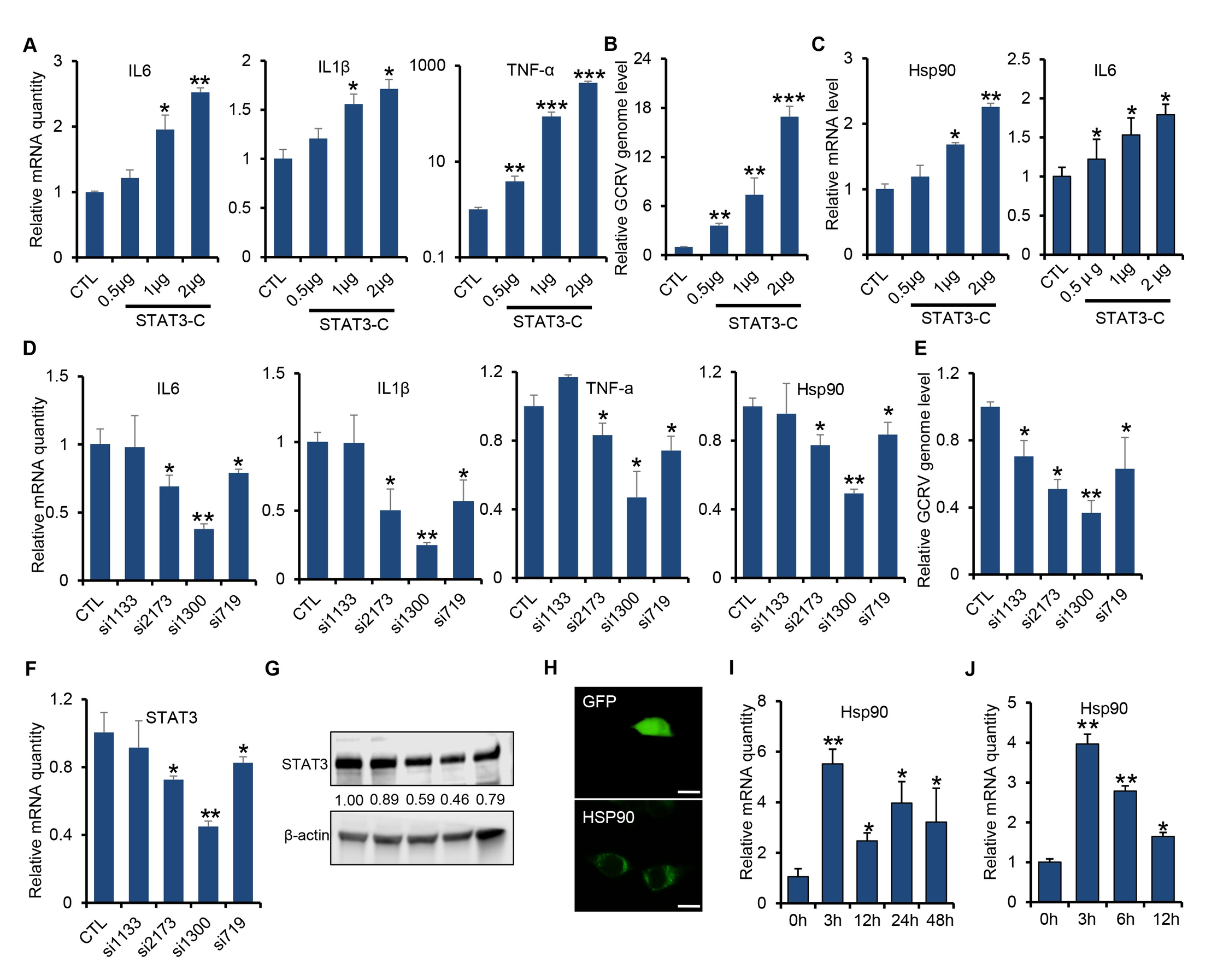

Supplement: S3 Fig — (A) CIK cells transfected with different doses of STAT3-C plasmids were prepared to quantify the relative expression of IL6, IL1β, and TNF-α by RT-PCR analysis. (B) CIK cells transfected with different doses of STAT3-C plasmids were infected with GCRV (MOI = 5) for 8 hours and prepared to quantify the relative GCRV genome entry by RT-PCR analysis. (C) 293T cells transfected with different doses of STAT3-C plasmids were prepared to quantify the relative expression of IL6 and HSP90 by RT-PCR analysis. (D-F) CIK cells transfected with four different STAT3 siRNAs were infected with GCRV (MOI = 5) for 8 hours and prepared to quantify the transcription of IL6, IL1β, and TNF-α, and HSP90 (D) and the relative GCRV genome entry (E) by RT-PCR analysis. The STAT3 knockdown efficiency was analyzed by RT-PCR (F) and Western blotting (G). (H) CIK cells transfected with pEGFP-HSP90 were prepared to perform fluorescence analysis. pEGFP-N1 served as a control. (I-J) Gills of grass carp (I) or CIK cells (J) under temperature-switch treatment from 18°C to 28°C were prepared to quantify the relative expression of HSP90 by RT-PCR. Data were presented as mean ± SD from three independent experiments. Statistical analysis was performed using one-way ANOVA between different groups and the asterisk (*) indicates significant differences between groups. *p<0.05, **p<0.01, ***p<0.001. (TIF) [file ppat.1011320.s003.tif]

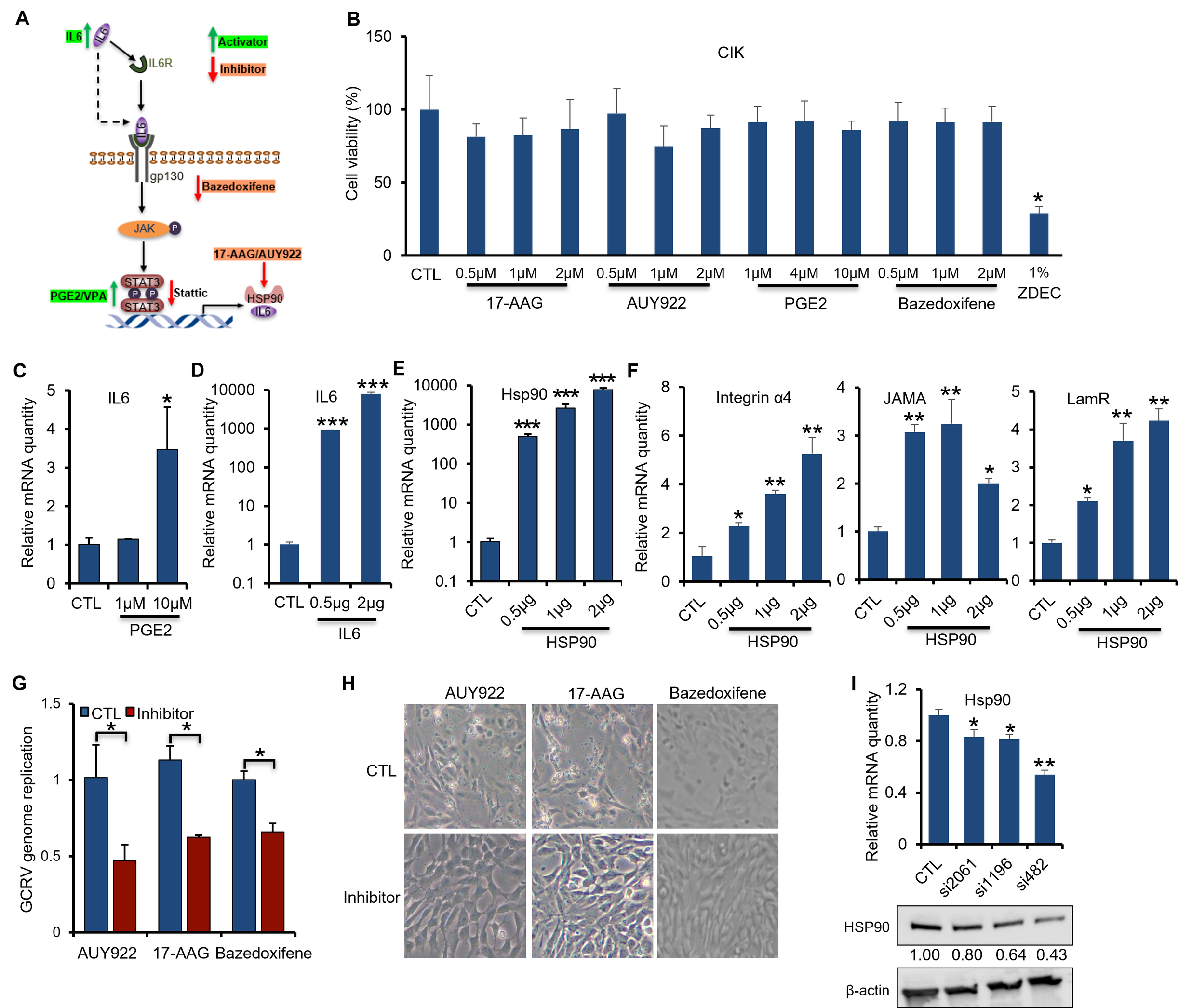

Supplement: S4 Fig — (A) Relevant activators or inhibitors involved in the IL6-STAT3-HSP90 signaling axis were utilized in this study. (B) CIK cells treated with different doses of drugs were prepared to analyze the cell viability by trypan blue staining. ZDEC served as a positive control. (C) CIK cells pre-treated with different doses of PGE2 were prepared to analyze the relative transcription of IL6 by RT-PCR. (D) CIK cells transfected with different doses of grass carp IL6 plasmids were prepared to quantify the relative transcription of IL6 by RT-PCR. (E-F) CIK cells transfected with different doses of HSP90 plasmids were infected with GCRV (MOI = 5) at 28°C for 1 h and harvested to quantify the relative transcription of HSP90, integrin α4, JAMA, and LamR by RT-PCR. (G) CIK cells treated with AUY922, 17-AAG, or bazedoxifene were infected with GCRV at 28°C for 8 h and harvested to quantify the relative viral genome replication by RT-PCR. (H) CIK cells from S4G Fig were prepared for CPE monitoring by optical microscope analysis. (I) CIK cells transfected with three different HSP90 siRNAs were infected with GCRV (MOI = 5) for 1 hour and prepared to quantify the relative transcription and protein translation of HSP90. Data were presented as mean ± SD from three independent experiments. Statistical analysis was performed using one-way ANOVA between different groups and the asterisk (*) indicates significant differences between groups. *p<0.05, **p<0.01, ***p<0.001. (TIF) [file ppat.1011320.s004.tif]

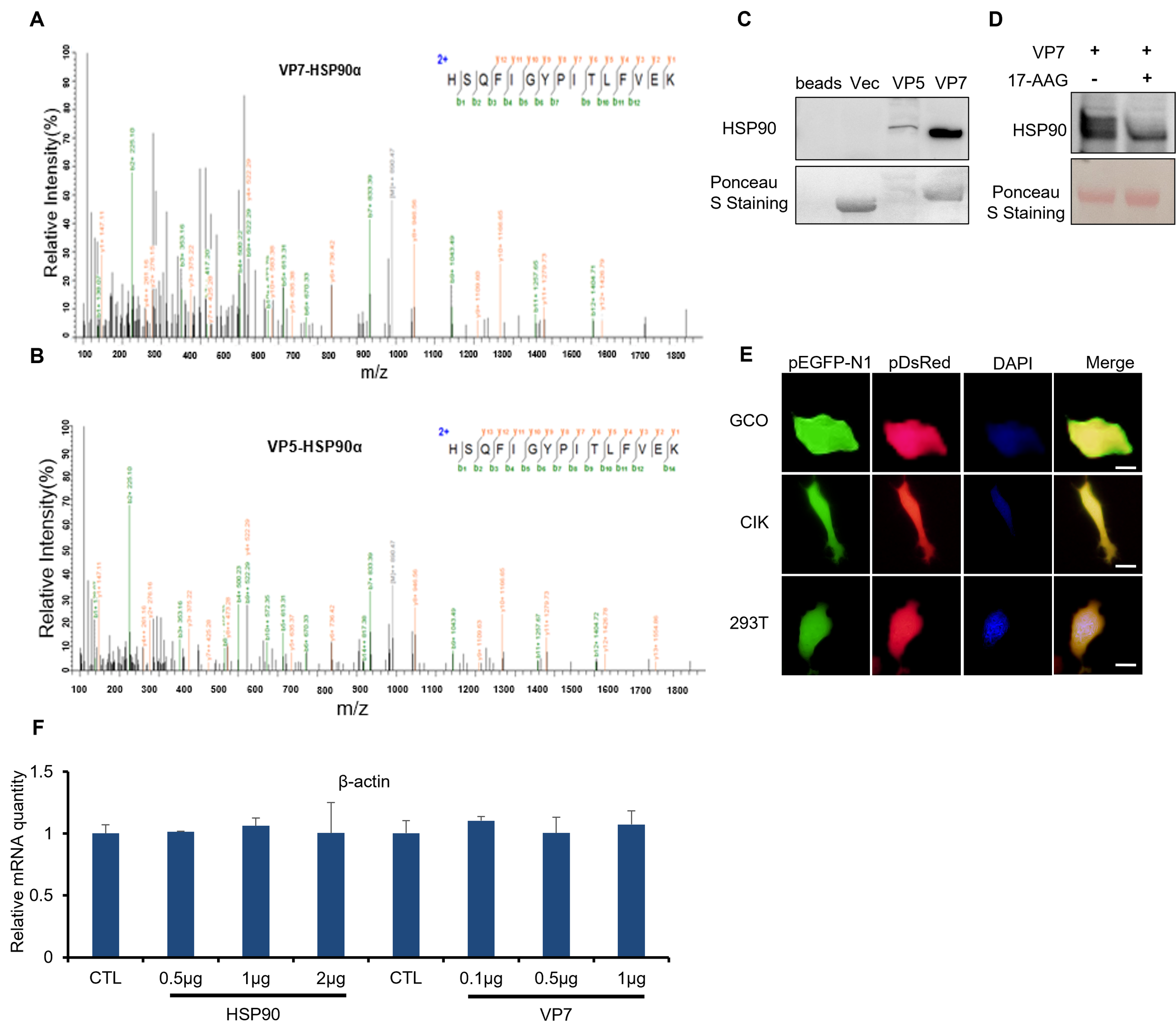

Supplement: S5 Fig — (A-B) CIK cell lysates were incubated with VP5-HIS-EGFP coupled with Ni-beads, and the pulldown complex was then prepared for mass spectrometry analysis. The mass/charge ratio (m/z) spectrum peptide analysis identified HSP90 as a binding candidate of VP7 (A) and VP5 (B). (C) CIK cell lysates were incubated with purified his-tagged VP5/VP7 protein-coupled by Ni-beads, and the precipitates from the pulldown complex were prepared to confirm the interaction between VP5/VP7 and endogenous HSP90 in CIK cells. (D) CIK cells treated with 17-AAG were prepared to examine the interaction between purified his-tagged VP7 and endogenous HSP90 from CIK cell lysates by pulldown analysis. (E) GCO, CIK, or 293T cells stably expressing tagged pEGFP-N1 and pDsRed were prepared to perform immunofluorescence analysis. Scale bars: 20 μm. (F) CIK cells transfected withdifferent doses of HSP90 or VP7 plasmids were prepared to quantify the relative transcription of β-actin with GAPDH as the reference gene. (TIF) [file ppat.1011320.s005.tif]

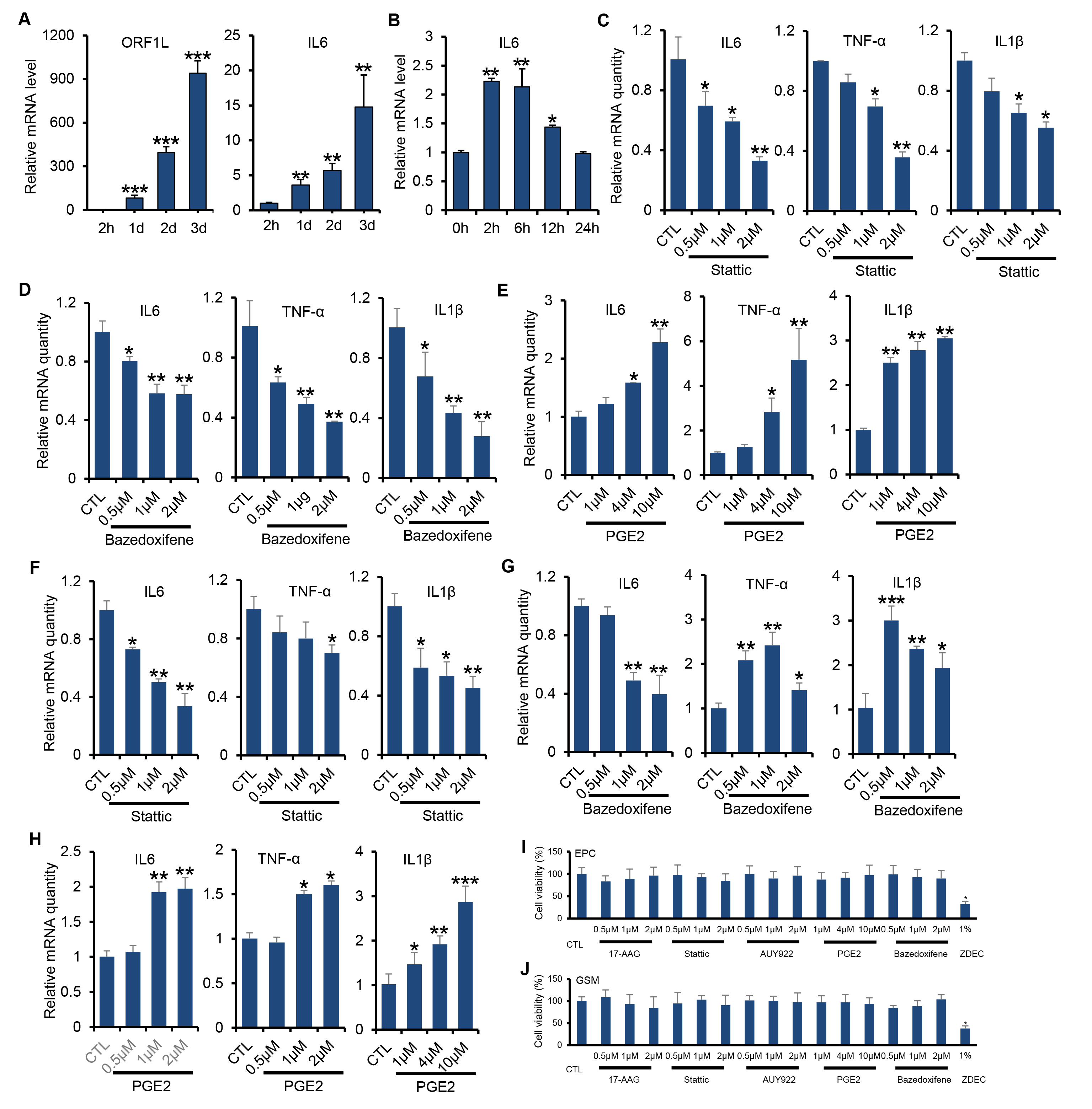

Supplement: S6 Fig — (A) CCB cells under lytic infection of KHV at 22°C were prepared to quantify the relative transcription of an early gene of KHV ORF1L and IL6 by RT-PCR. (B) Gills from Koi (average weight 10 g) under temperature-switch treatment from 10°C to 20°C for different time points were harvested to analyze the relative expression of IL6 by RT-PCR. (C-E) CCB cells were treated with different doses of Stattic (C), bazedoxifene (D), or PGE2 (E) for 2 hours, followed by KHV infection for 1 hour. The cells were then harvested to quantify the relative transcription of IL6, TNF-α, and IL1β by RT-PCR. (F-H) EPC cells were treated with different doses of Stattic (C), bazedoxifene (D), or PGE2 (E) for 2 hours, followed by SVCV infection for 1 hour. The cells were then harvested to quantify the relative transcription of IL6, TNF-α, and IL1β by RT-PCR. (I-J) EPC or GSM cells pre-treated with different doses of drugs for 2 hours were prepared to analyze the cell viability by trypan blue staining. ZDEC served as a positive control. Data were presented as mean ± SD from three independent experiments. Statistical analysis was performed using one-way ANOVA between different groups and the asterisk (*) indicates significant differences between groups. *p<0.05, **p<0.01, ***p<0.001. (TIF) [file ppat.1011320.s006.tif]
